# Follicular metabolic alterations are associated with obesity in mares and can be mitigated by dietary supplementation

**DOI:** 10.1038/s41598-024-58323-0

**Published:** 2024-03-30

**Authors:** Giovana D. Catandi, Kyle J. Fresa, Ming-Hao Cheng, Luke A. Whitcomb, Corey D. Broeckling, Thomas W. Chen, Adam J. Chicco, Elaine M. Carnevale

**Affiliations:** 1 Equine Reproduction Laboratory, Department of Biomedical Sciences, Colorado State University, 3101 Rampart Road, Fort Collins, CO 80521 USA; 2 Department of Biomedical Sciences, Colorado State University, Fort Collins, CO 80523 USA; 3 Department of Electrical and Computer Engineering, Colorado State University, Fort Collins, CO 80523 USA; 4 Proteomics and Metabolomics Facility, Colorado State University, Fort Collins, CO 80523 USA; 5 School of Biomedical Engineering, Colorado State University, Fort Collins, CO 80523 USA; 6 Department of Veterinary Clinical Sciences, Oklahoma State University, Stillwater, OK 74078 USA

**Keywords:** Developmental biology, Metabolic disorders, Reproductive disorders

## Abstract

Obesity is a growing concern in human and equine populations, predisposing to metabolic pathologies and reproductive disturbances. Cellular lipid accumulation and mitochondrial dysfunction play an important role in the pathologic consequences of obesity, which may be mitigated by dietary interventions targeting these processes. We hypothesized that obesity in the mare promotes follicular lipid accumulation and altered mitochondrial function of oocytes and granulosa cells, potentially contributing to impaired fertility in this population. We also predicted that these effects could be mitigated by dietary supplementation with a combination of targeted nutrients to improve follicular cell metabolism. Twenty mares were grouped as: Normal Weight [NW, n = 6, body condition score (BCS) 5.7 ± 0.3], Obese (OB, n = 7, BCS 7.7 ± 0.2), and Obese Diet Supplemented (OBD, n = 7, BCS 7.7 ± 0.2), and fed specific feed regimens for ≥ 6 weeks before sampling. Granulosa cells, follicular fluid, and cumulus-oocyte complexes were collected from follicles ≥ 35 mm during estrus and after induction of maturation. Obesity promoted several mitochondrial metabolic disturbances in granulosa cells, reduced L-carnitine availability in the follicle, promoted lipid accumulation in cumulus cells and oocytes, and increased basal oocyte metabolism. Diet supplementation of a complex nutrient mixture mitigated most of the metabolic changes in the follicles of obese mares, resulting in parameters similar to NW mares. In conclusion, obesity disturbs the equine ovarian follicle by promoting lipid accumulation and altering mitochondrial function. These effects may be partially mitigated with targeted nutritional intervention, thereby potentially improving fertility outcomes in the obese female.

## Introduction

Obesity is a growing public health concern in the human population and is linked with metabolic disturbances and a predisposition to infertility^1^. Obese women undergoing clinical assisted reproduction technology (ART) procedures have reduced embryo development and lower pregnancy and live birth rates in comparison to normal-weight women^2^. Among the reproductive alterations promoted by obesity, changes in the follicular environment and oocyte are numerous and likely contribute to reproductive complications^3^. Granulosa cells line the ovarian follicle and play essential roles in metabolism and transport of nutrients from the systemic circulation to the follicular fluid, providing an appropriate local environment for the developing oocyte. Cumulus cells are a specialized type of granulosa cells that directly surround and nurture the oocyte through cellular communications^4^. Obese women tend to have more lipids in their ovarian follicles^3^. In vivo and in vitro research models have demonstrated that prolonged exposure to elevated lipids lead to impaired oocyte developmental potential and could contribute to transgenerational transmission of metabolic diseases, as mitochondria from the oocyte give rise to all mitochondria in future offspring^3,5,6^.

Obesity is also observed in the equine population, with a prevalence of up to 50% in different regions of the USA^7–10^. Similar to humans, equine obesity increases the propensity for metabolic pathologies, particularly insulin dysregulation and metabolic syndrome^11,12^. Reproductive disturbances and alterations have been associated with obesity in the mare^13,14^, including lipid accumulation in the follicle and oocyte^15^. These can contribute to life-long effects on offspring health, as metabolic and inflammatory changes are observed in foals from obese mares^16,17^. Although fetal exposure to obesogenic signals during gestation contributes to developmental programming of metabolic diseases, there is growing evidence that preconception alterations in the oocyte establish transgenerational transmission of obesity and insulin resistance^6^. The mare is considered a strong animal model for investigation of the effects of maternal conditions on human reproduction due to several important similarities in reproductive physiology, including hormonal signaling, ovulatory patterns, and ART procedures routinely performed clinically^18–21^. Additionally, the large equine preovulatory follicle allows abundant and relatively easy collection of follicular fluid and cells^22^.

Using the mare model, we have previously demonstrated the negative effects of advanced maternal age on oocyte aerobic and anaerobic metabolism^23–25^, quantified through microsensor measurement of, respectively, oxygen consumption rate (OCR) and extracellular acidification rate (ECAR)^26,27^. In a follow up study, we demonstrated that supplementing older mares with a combination of nutrients designed to promote gastrointestinal wellness and cellular metabolism improved oocyte metabolism, reduced oocyte lipid accumulation, and increased embryonic development after intracytoplasmic sperm injection (ICSI)^28–30^. These findings indicate that short-term dietary interventions are a feasible approach to target age-induced changes in the ovarian follicular environment and oocyte, ultimately improving fertility outcomes from aged mares.

Alterations in oocytes promoted by maternal obesity seem to be associated with lipid overload in the follicle; however, this ultimately leads to mitochondrial dysfunction and oxidative stress in granulosa cells and oocytes^3,31^. Studies in humans and rodents demonstrate potential beneficial effects of certain dietary additives on cellular metabolism. Among studied feed ingredients, L-carnitine and chromium, alone or in combination, have been shown to improve insulin sensitivity, reduce circulating lipid concentrations, and attenuate oxidative stress in obese mice and women^32–36^. Obesity-induced mitochondrial dysfunction is associated with reduced free L-carnitine availability, which can be restored by diet supplementation^32,37^. L-carnitine is a mitochondrial co-factor essential for oxidation of fatty acids and regulation of pyruvate oxidation^37^. In obese mammals, excessive circulating lipids bind to free L-carnitine, leading to formation of long-chain acylcarnitines that efflux from mitochondria and accumulate in body fluids. This results in a depletion of free L-carnitine inside mitochondria and reduces the mitochondrial capacity to metabolize substrates such as fatty acids and pyruvate^37^. Although diet recommendations are available for women undergoing ART procedures, specific recommendations especially for obese women, are still unclear, and similar recommendations are even more scarce for mares.

The objectives of the present study were to characterize changes in lipid profiles and metabolic function of cells in the ovarian follicle associated with obesity in mares, as well as to assess the potential of dietary supplementation to mitigate these changes. We hypothesized that obesity promotes lipid accumulation in the ovarian follicle and negatively affects mitochondrial metabolism in oocytes and granulosa cells. However, dietary supplementation with a targeted combination of nutrients, including L-carnitine, would improve cell metabolic function in the follicles of obese mares, ultimately contributing to follicle and oocyte viability and, potentially, offspring health.

## Results

### Morphometric measurements of mares

Mares were closely monitored and fed treatment diets for > 6 weeks prior to follicular sample collections. Mares in three treatment groups: Normal Weight (NW), Obese (OB) and Obese Diet Supplemented (OBD) were assessed at 2-week intervals for changes in body weight and for morphometric indicators of adiposity, including body condition score (BCS), percentage body fat, and cresty neck score (Supplementary Fig. S1). Throughout the study, morphometric measurements were mostly consistent within groups. Although no differences were noted for body weight among groups at any time point (NW: 530.0 ± 6.5, OB: 587.3 ± 8.0, OBD: 576.6 ± 6.0, P ≥ 0.1; Supplementary Fig. S1a), BCS and percentage body fat were greater in OB and OBD than NW throughout the study (respectively: NW: 5.6 ± 0.1, OB: 7.6 ± 0.1, OBD: 7.4 ± 0.1, P ≤ 0.009; 7.0 ± 0.3, OB: 13.3 ± 0.7, OBD: 12.2 ± 0.4, P ≤ 0.04; Supplementary Fig. S1b,c), and the same differences were noted in cresty neck score from week 6 to 12 (P ≤ 0.04; Supplementary Fig. S1d).

### Granulosa cell mitochondrial function and enzyme gene and protein expression

**Figure 1:**
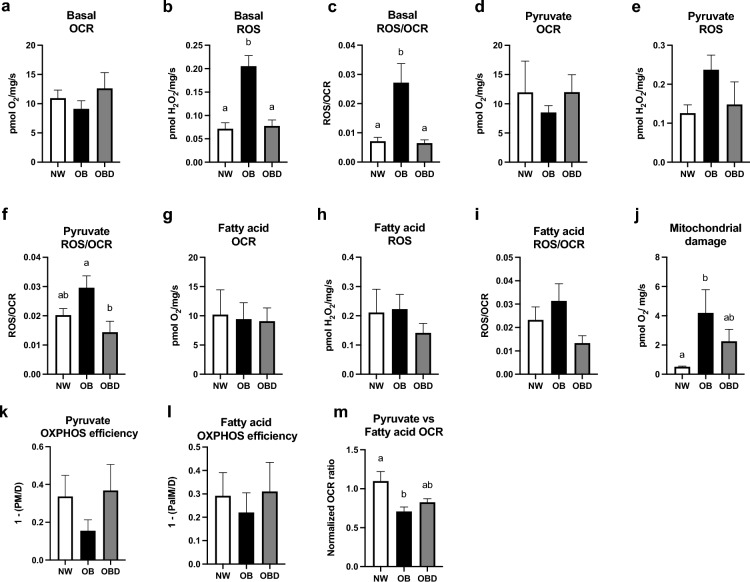
Effects of mare obesity and diet supplementation on granulosa cell mitochondrial function. Mitochondrial function of granulosa cells obtained from preovulatory follicles of normal-weight (NW, n = 6), obese (OB, n = 7) and obese diet supplemented (OBD, n = 6) mares after ≥ 6 weeks of supplementation, expressed as the basal rates of oxygen consumption (OCR) (**a**), H_2_O_2_ (ROS) release (**b**), and ROS release as a proportion of OCR (ROS/OCR) (**c**) measured from intact cells under basal conditions; the carbohydrate oxidative phosphorylation (OXPHOS)-linked OCR (**d**), ROS release (**e**), and ROS/OCR (**f**) measured in permeabilized cells energized with 1 mM malate, 5 mM pyruvate, and 2.5 mM ADP; and the fatty acid OXPHOS-linked OCR (**g**), ROS release (**h**), and ROS/OCR (**i**) measured in permeabilized cells energized with 1 mM malate, 0.05 mM palmitoylcarnitine, and ADP. Mitochondrial inner membrane damage assessed by the increase in maximal OXPHOS-linked OCR following the addition of cytochrome *c* (**j**). Indices of OXPHOS coupling efficiency [calculated as 1-(LEAK/OXPHOS OCR)] supported by pyruvate (**k**) and fatty acid (**l**) substrates. An index of carbohydrate versus fat oxidation capacity expressed as the pyruate:palmitoylcarnitine OCR ratio normalized to the maximal OXPHOS-linked rate as described in Methods (**m**). Graphs represent mean ± SEM. Different superscripts indicate differences among groups using one-way ANOVA with post-hoc Tukey’s multiple comparison tests, or Kruskal–Wallis tests, followed by Dunn’s multiple comparison tests (P < 0.05).

Oxygen consumption rate (OCR) and hydrogen peroxide (ROS) release rate were measured in intact cells without the addition of exogenous substrate (basal conditions), and in digitonin-permeabilized cells enabling delivery of saturating concentrations of pyruvate (5 mM) or fatty acid (0.05 mM palmitoylcarnitine) in the presence of malate (1 mM) and adenosine diphosphate (ADP 2.5 mM) to determine maximal rates of oxidative phosphorylation (OXPHOS)-linked OCR and ROS release supported by pyruvate or fatty acid oxidation, respectively. While basal OCR did not differ among groups (P = 0.4; Fig. 1a), basal rates of ROS release and ROS/OCR were greater in OB than NW and OBD (P ≤ 0.0005; Fig. 1b,c). Maximal OXPHOS-linked OCR and ROS production capacities were similar among groups for both pyruvate and palmitoylcarnitine (P ≥ 0.7; Fig. 1d,e,g,h). However, when normalized to OCR, ROS release during pyruvate-supported OXPHOS was greater in OB than OBD (P = 0.04; Fig. 1f), although not during palmitoylcarnitine-supported OXPHOS (P ≥ 0.2; Fig. 1i). Mitochondrial inner membrane damage tended to be greater in granulosa cells from OB than NW mares (P = 0.06), indicated by a greater increase in OXPHOS-linked OCR following the addition of cytochrome *c,* with no significant difference noted between OBD and NW (P ≥ 0.1; Fig. 1j). Indices of OXPHOS coupling efficiency were calculated from OCR supported by pyruvate or palmitoylcarnitine in the absence of ADP (LEAK) and presence of ADP (OXPHOS) as [1-(LEAK/OXPHOS)], which appeared to reflect impaired coupling efficiency in OB when compared to the other groups, but this did not reach statistical significance (P ≥ 0.1; Fig. 1k,l). Finally, mitochondrial substrate preference, calculated as the OCR ratio of pyruvate:palmitoylcarnitine oxidation normalized to the maximal OXPHOS rate (OCR supported by substrates + succinate) of each sample, was greater in NW than OB (P = 0.02; Fig. 1m), reflecting a greater relative capacity of OB mitochondria to oxidize fatty acids over carbohydrates when compared to NW.

**Figure 2:**
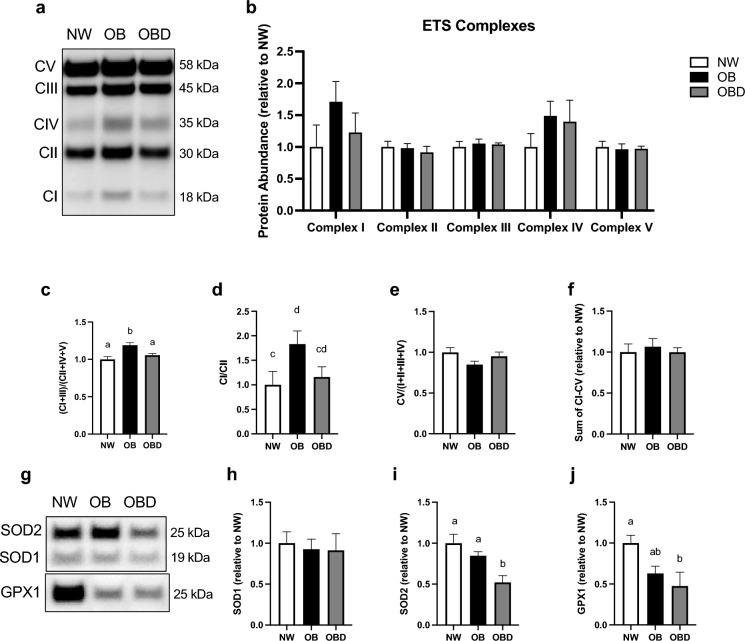
Effects of mare obesity and diet supplements on granulosa cell expression of mitochondrial proteins. Expression of mitochondrial complexes and antioxidant proteins in granulosa cells obtained from preovulatory follicles of normal weight (NW, n = 5), obese (OB, n = 5) and obese diet supplemented (OBD, n = 5) mares after ≥ 6 weeks of supplementation; data are presented as fold changes relative to the control group (NW): (**a**) representative western blot of electron transport system complexes I–V, (**b**) expression of subunits from each of the electron transport system complexes, (**c**) ROS producing complexes (I and III) relative to other complexes, (**d**) complex I relative to complex II, (**e**) complex V (ATP synthase) relative to other complexes (II, IV and V), and (**f**) the sum of all five complexes; (**g**) representative western blot of superoxide dismutase 2 (SOD2), superoxide dismutase 1 (SOD1), and glutathione peroxidase 1 (GPX1), (**h**) protein expression of SOD1, (**i**) SOD2, and (**j**) GPX1. Graphs represent mean ± SEM. Different superscripts indicate differences between groups at P < 0.05 (^ab^) and P < 0.1 (^cd^) using one-way ANOVA with post-hoc Tukey’s multiple comparison tests.

The relative protein abundance of each of the five electron transport complexes (I–V) in granulosa cells was not significantly different across groups (P ≥ 0.4; Fig. 2b); however, the expression of ROS producing complexes (I and III) relative to non-ROS producing complexes (II, IV and V) was greater in granulosa cells from OB when compared to NW and OBD (P ≤ 0.03; Fig. 2c). Proportional protein abundance of complex I over complex II also tended to be greater in granulosa cells from OB than NW (P = 0.08), while OBD was not different from the other groups (P ≥ 0.2; Fig. 2d). Protein abundance of the ATP producing complex V (ATP synthase) in relation to abundance of all other complexes (I – IV) was not affected by mare group (P ≥ 0.1; Fig. 2e), nor was the total protein expression of all electron transport system complexes (P ≥ 0.8; Fig. 2f). Expression of the cytosolic superoxide dismutase isoform 1 was not different among groups (P ≥ 0.9; Fig. 2h), while the mitochondrial superoxide dismutase isoform 2 was lower in granulosa cells from OBD than NW and OB (P ≤ 0.04; Fig. 2i). Similarly, glutathione peroxidase 1 protein expression was lower in OBD when compared to NW (P = 0.03), but OB was not different from the other groups (P ≥ 0.1; Fig. 2j).

Gene expression specific to pathways of interest resulted in no differences being observed among groups for most of the genes assessed (P ≥ 0.1; Supplementary Fig. S2a–m), with the exception that *CYP19A1* mRNA abundance was greater in granulosa cells from OB when compared to NW and OBD (P ≤ 0.004; Supplementary Fig. S2n).

### Follicular fluid lipid and acylcarnitine abundance

**Figure 3:**
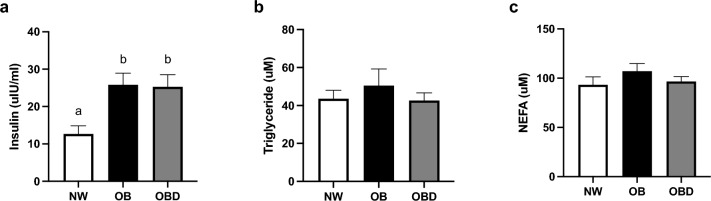
Effects of mare obesity and diet supplementation on follicular fluid insulin and lipid concentrations. Concentration of insulin and different lipid species in follicular fluid obtained from preovulatory follicles of normal-weight (NW, n = 6), obese (OB, n = 7) and obese diet supplemented (OBD, n = 6) mares after ≥ 6 weeks of supplementation: (**a**) insulin , (**b**) triglycerides, and (**c**) non-esterified fatty acids. Graphs represent mean ± SEM. Different superscripts indicate difference (P < 0.05) among groups using one-way ANOVA with post-hoc Tukey’s multiple comparison tests.

**Figure 4:**
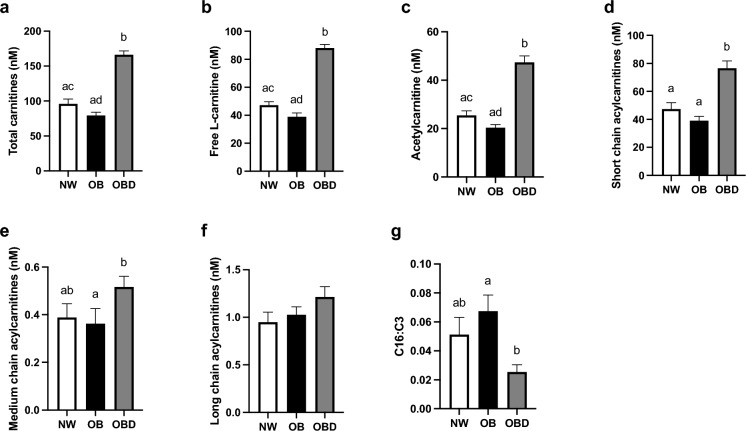
Effects of mare obesity and diet supplementation on follicular fluid concentration of acylcarnitines. Concentration of acylcarnitine species in follicular fluid obtained from preovulatory follicles of normal-weight (NW, n = 6), obese (OB, n = 7) and obese diet supplemented (OBD, n = 6) mares after ≥ 6 weeks of supplementation: (**a**) total carnitine species (TC), (**b**) free L-carnitine (FC), (**c**) acetyl-carnitine (C2AC), (**d**) short-chain acylcarnitines (SCAC), (**e**) medium-chain acylcarnitines (MCAC), (**f**) long-chain acylcarnitines (LCAC), and (**g**) ratio of C16:C3 aceylcarnitines, indicative of complete β-oxidation rate. Graphs represent mean ± SEM. Different superscripts indicate difference (^ab^P < 0.05) or tendency for difference (^cd^P < 0.1) between groups using one-way ANOVA with post-hoc Tukey’s multiple comparison tests.

The concentration of insulin in follicular fluid was higher in both obese groups (OB and OBD) when compared to NW (P ≤ 0.02; Fig. 3a). Triglyceride and non-esterified fatty acid concentrations did not differ among groups (P ≥ 0.4; Fig. 3b,c). When comparing NW to OB, mare obesity did not significantly affect any short-, mid- or long-chain individual acylcarnitine species (P ≥ 0.1; Table 1); however, diet supplementation to obese mares increased follicular concentrations of several acylcarnitines, mainly short-chain, in comparison to other groups (P ≤ 0.03; Table 1). Abundance of total carnitines (sum of all acylcarnitine species and free L-carnitine), free L-carnitine, and acetylcarnitine tended to be less in OB than NW (P = 0.09) and were greater in OBD than both other groups (P < 0.0001; Fig. 4a–c). Follicular concentration of the sum of all short-chain acylcarnitines was greater in OBD than NW and OB (P ≤ 0.0007; Fig. 4d). Total concentration of mid-chain acylcarnitines was greater in follicular fluid from OBD than OB (P = 0.05) and similar to NW (P = 0.3); OB and NW were not different (P > 0.99; Fig. 4e). No group differences were noted for total long-chain acylcarnitine concentrations in follicular fluid (P ≥ 0.2; Fig. 4f). An elevated ratio between palmitoylcarnitine (C16) and propionylcarnitine (C3) is indicative of ineffective β-oxidation^38^; the ratio was lower in OBD than OB (P = 0.02), with both similar to NW (P ≥ 0.2; Fig. 4g).

**Table 1:** Concentration of individual acylcarnitine species (nM) in follicular fluid obtained from preovulatory follicles of normal-weight (NW, n = 6), obese (OB, n = 7) and obese diet supplemented (OBD, n = 6) mares after ≥ 6 weeks of supplementation.

	NW	OB	OBD
Short-chain acylcarnitines			
Acetyl (C2)	25.48 ± 1.91^ac^	20.37 ± 1.00^ad^	47.41 ± 2.67^b^
Propionyl (C3)	3.76 ± 0.71	2.35 ± 0.25	6.97 ± 0.75
Succinyl (C4-DC)	10.89 ± 1.96	11.39 ± 1.84	10.18 ± 1.78
Hydroxybutyryl (C4-OH)	1.93 ± 0.36	1.23 ± 0.30	2.48 ± 0.66
Butanoyl (C4)	3.55 ± 0.70^ab^	2.44 ± 0.53^a^	6.46 ± 1.00^b^
Hydroxyisovaleryl (C5-OH)	0.18 ± 0.02	0.18 ± 0.03	0.27 ± 0.05
Isovaleryl (C5)	1.62 ± 0.15^a^	1.12 ± 0.12^a^	2.78 ± 0.11^b^
Medium-chain acylcarnitines			
Adipyl (C6-DC)	0.27 ± 0.04	0.26 ± 0.05	0.35 ± 0.03
Hexanoyl (C6)	0.08 ± 0.01	0.06 ± 0.01	0.12 ± 0.02
Decanoyl (C10)	0.02 ± 0.001	0.02 ± 0.01	0.03 ± 0.003
Dodecanoyl (C12)	0.02 ± 0.002	0.02 ± 0.003	0.02 ± 0.002
Long-chain acylcarnitines			
Tetradecenoyl (C14:1)	0.05 ± 0.01	0.04 ± 0.01	0.07 ± 0.01
Tetradecanoyl (C14)	0.02 ± 0.02	0.02 ± 0.003	0.03 ± 0.003
Hexadecenoyl (C16:1)	0.07 ± 0.01	0.07 ± 0.01	0.09 ± 0.02
Palmitoyl (C16)	0.17 ± 0.03	0.15 ± 0.02	0.16 ± 0.02
Linoleoyl (C18:2)	0.12 ± 0.03^a^	0.25 ± 0.02^ab^	0.37 ± 0.04^b^
Octadecenoyl (C18:1)	0.43 ± 0.07	0.50 ± 0.07	0.50 ± 0.05

Results are presented as mean ± SEM. Different superscripts within the same row indicate difference (^ab^P < 0.05) or tendency for difference (^cd^P < 0.1) between groups using one-way ANOVA with post-hoc Tukey’s multiple comparison tests.

### Cumulus cell and oocyte lipid profiles

**Figure 5:**
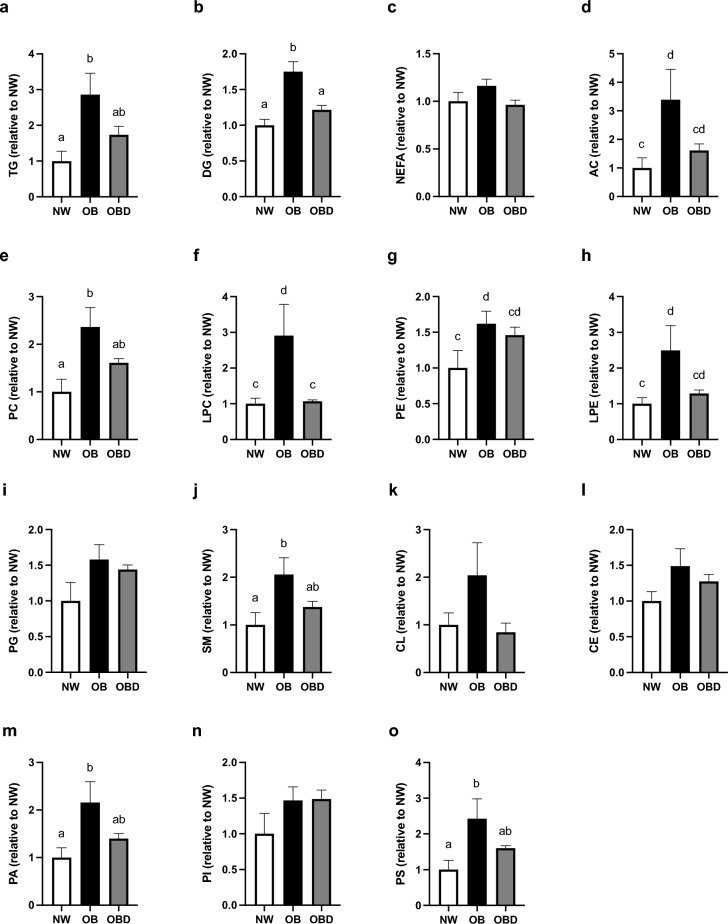
Effects of mare obesity and diet supplementation on cumulus cell lipid abundance. Relative abundance of different lipid species categories in cumulus cells obtained from preovulatory follicles of normal-weight (NW, n = 5), obese (OB, n = 7) and obese, diet-supplemented (OBD, n = 6) mares after ≥ 6 weeks of supplementation, calculated as fold change relatively to NW: (**a**) triglycerides (TG), (**b**) diacylglycerols (DG), (**c**) non-esterified fatty acids (NEFA), (**d**) acylcarnitines (AC), (**e**) phosphatidylcholines (PC), (**f**) lyso-phosphatidylcholines (LPC), (**g**) phosphatidylethanolamines (PE), (**h**) lyso-phosphatidyletanolamines (LPE), (**i**) phosphatidylglycerols (PG), (**j**) sphingomyelins (SM), (**k**) cardiolipins (CL), (**l**) cholesteryl-esters (CE), (**m**) phosphatidic acids (PA), (**n**) phosphatidylinositols (PI), and (**o**) phosphatidylserines (PS). Graphs represent mean ± SEM. Different superscripts indicate difference (^ab^P < 0.05) or tendency for difference (^cd^P < 0.1) among groups using one-way ANOVA with post-hoc Tukey’s multiple comparison tests.

A total of 1,267 lipid species were identified in cumulus cells, from which 87 differed in abundance among the three groups (Supplementary Table 1). Of the 87 differing lipids, triglyceride was the most represented lipid class (44%). Lipid species were compared among groups as fold changes relatively to the control group (NW), and the mean fold change for each lipid class was compared among groups. We observed a general trend for lipid classes to be more abundant in cumulus cells from OB than NW and OBD (Fig. 5). The normalized abundance of triglycerides, acylcarnitines, phosphatidylcholines, phosphatidylethanolamines, lyso-phosphatidyletanolamines, sphingomyelins, phosphatidic acids and phosphatidylserines were significantly greater or tended to be greater in cumulus cells from OB than NW (P ≤ 0.08); OBD was similar to NW and OB (P ≥ 0.2; Fig. 5a,d,e,g,h,j,m,o). Normalized abundance of diacylglycerols and lyso-phosphatidylcholines were significantly greater or tended to be greater in OB than NW and OBD (P ≤ 0.09; Fig. 5b,f). No significant differences were observed among groups for normalized abundance of non-esterified fatty acids, phosphatidylglycerols, cardiolipins, cholesteryl-esters, and phosphatidylinositols (P ≥ 0.2; Fig. 5c,i,k,l,n).

**Figure 6:**
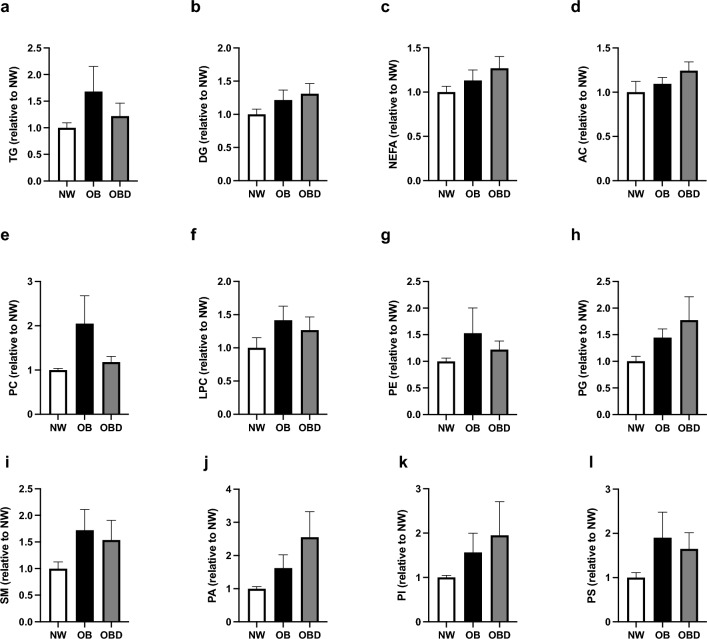
Effects of mare obesity and diet supplementation on oocyte lipid abundance. Relative abundance of different lipid species categories in oocytes obtained from preovulatory follicles of normal-weight (NW, n = 5), obese (OB, n = 7) and obese diet supplemented (OBD, n = 6) mares after ≥ 6 weeks of supplementation, calculated as fold change relatively to NW: (**a**) triglycerides (TG), (**b**) diacylglycerols (DG), (**c**) non-esterified fatty acids (NEFA), (**d**) acylcarnitines (AC), (**e**) phosphatidylcholines (PC), (**f**) lyso-phosphatidylcholines (LPC), (**g**) phosphatidylethanolamines (PE), (**h**) phosphatidylglycerols (PG), (**i**) sphingomyelins (SM), (**j**) phosphatidic acids (PA), (**k**) phosphatidylinositols (PI), and (**l**) phosphatidylserines (PS). Graphs represent mean ± SEM. Differences were not significant (P ≥ 0.2) among groups.

In individual oocytes, a total of 335 lipid species were identified, from which 19 differed in abundance among groups (Supplementary Table 2). Similar to observations in cumulus cells, the most represented lipid class that differed among groups was triglyceride (42% of the individual lipid species that differed among groups). Fold change analyses for lipid classes were performed in oocytes, and no differences were observed for the identified lipid classes (P ≥ 0.2; Fig. 6a–l), although many of the lipids appeared to have a similar distribution for oocytes and cumulus cells.

### Oocyte metabolic function

**Figure 7:**
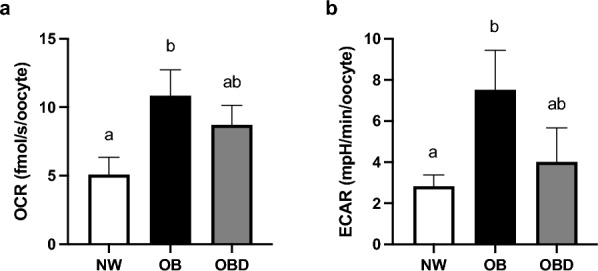
Effects of mare obesity and diet supplementation on oocyte metabolism. Basal metabolic function of oocytes obtained from preovulatory follicles of normal weight (NW, n = 6), obese (OB, n = 7) and obese diet supplemented (OBD, n = 6) mares: (**a**) basal aerobic metabolism, measured as oxygen consumption rate (OCR) and (**b**) basal anaerobic metabolism, measured as extracellular acidification rate (ECAR). Graphs represent mean ± SEM. Different superscripts indicate difference among groups using one-way ANOVA with post-hoc Tukey’s multiple comparison tests (P < 0.05).

Mature oocytes were denuded of cumulus cells and assayed for basal aerobic and anaerobic metabolism, measured respectively as OCR and ECAR, using microsensors. Oocyte OCR was higher in OB than NW (P = 0.04), and not different in OBD compared to NW and OB (P ≥ 0.3; Fig. 7a). Similar outcomes were noted for anaerobic metabolism, with higher ECAR in oocytes from OB relative to NW (P = 0.05), and no difference between OBD and NW or OB (P ≥ 0.2; Fig. 7b).

## Discussion

Maternal obesity negatively affects fertility outcomes and may contribute to transgenerational transmission of metabolic diseases^3,6^. Using several animal models, researchers have elucidated that obesity-induced alterations in the oocyte are mainly associated with lipid accumulation and the resultant mitochondrial dysfunction and oxidative stress^5,15,39–42^. Improving oocyte mitochondrial function may help ameliorate oocyte and embryo quality from obese females and limit propagation of dysfunctional mitochondria to subsequent generations, as mitochondria from the oocyte give rise to all mitochondria in offspring^6^. Diet interventions are a feasible in vivo approach to potentially improve female fertility. The present study was designed to examine the effects of mare obesity on metabolism of cells in the ovarian follicle and the potential of nutritional interventions to improve the follicular environment, specifically in terms of lipid accumulation and cellular metabolic function. The ovarian follicle creates a microenvironment for the developing oocyte, with the potential to alter the amount and type of metabolic substrates which are provided to the oocyte. The large size (approximately 45 mm in diameter prior to ovulation) of the equine follicle and the similar maturation timeline to the human follicle^43^ provide the potential to explore the effects of excess adiposity on the ovarian follicle and potential treatment mechanisms in vivo.

While increased follicular lipid concentrations are generally associated with obesity in women^44–46^, conflicting results have been reported^47,48^, and less is known about mares. In a previous study, we reported a tendency for increased triglyceride concentration in the follicular fluid from obese when compared to normal-weight mares, as well as greater abundance of stearic and linoleic acids, although overall concentration of non-esterified fatty acids were not assessed^15^. In the present study, no differences in triglyceride or non-esterified fatty acid concentrations were observed, although more detailed assays, such as mass spectrometry utilized in our previous study, could have identified alterations in the fatty acid profile that may have contributed to observed outcomes. Studies that utilized similar methodology as performed in this study, reported no differences for triglyceride and non-esterified fatty acid concentrations in plasma from normal-weight and obese pregnant mares^17^; or in mature horses after dietary induction of obesity^49^.

Mice and in vitro bovine models have demonstrated that exposure to high lipid concentrations during oocyte maturation lead to lipid accumulation in cumulus cells^40,50,51^. Cumulus cell lipid accumulation is not always directly reflective of oocyte lipid accumulation, and there is evidence that the cumulus cell layer protects the oocyte from lipotoxic effects *in vitro*^51^*.* Associated with mare obesity, we observed an increased abundance of several lipid categories within cumulus cells, of which triglycerides were the most represented, as observed in the bovine in vitro model^52^. Supportive data was reported by our group with increased gene expression of perilipin-2, which stimulates accumulation of lipid droplets, in cumulus cells from obese when compared to normal-weight mares^15^. Abundance of diacylglycerols, phosphatidylethanolamines and phosphatidylserines in cumulus cells of women undergoing ART procedures have been linked with prediction of negative pregnancy outcome^53,54^, and these lipids were found in greater abundance in cumulus cells from obese than normal-weight mares in the present study. However, significant differences were not observed in oocytes. This potentially demonstrates a protective function of cumulus cells to prevent oocyte lipid accumulation, although the findings could also reflect a limited length of obesity and/or limited sample size. Additionally, advanced maternal age reduces abundance of transzonal projections in mice^55^ and potentially in mares^56^, which could contribute to limiting transfer of lipids from cumulus cells to oocytes as many of the mares in the present study were older. However, among lipid species that differed in oocytes between the groups, triglycerides were again the most represented, implying some degree of oocyte lipid accumulation promoted by obesity; this was reported previously in obese mares with increased abundance of some triglyceride species^15^. Equine oocytes are dark in appearance and are thought to be lipid laden, similar to pig oocytes^57^; although the effects of excessive lipid accumulation on equine oocyte developmental potential remains to be determined.

L-carnitine and acylcarnitines directly participate in mitochondrial metabolism by serving as substrates to essential mitochondrial enzymes^58^. Systemically, l-carnitine insufficiency, as promoted by obesity-related lipotoxicity, appears to link adiposity and insulin resistance^58,59^. In humans and rodent models, excessive lipids entering mitochondria form disproportionate quantities of long-chain acylcarnitines, which can cross membranes and accumulate in cells and circulation^58^. Whether maternal obesity promotes l-carnitine insufficiency in cells in the ovarian follicle remains unclear, but obese women have increased concentrations of long-chain acylcarnitines in their follicles when compared to normal-weight women^38^. This was not observed in the present study, in which follicular concentrations of individual or total long-chain acylcarnitines were not affected by mare obesity. Total mid- or short-chain acylcarnitines were also not affected. However, the tendency for lower total carnitines, free L-carnitine, and acetyl-carnitine in follicular fluid from OB when compared to NW suggests that L-carnitine insufficiency may limit carnitine acetyl-transferase activity. This could impair follicular cell mitochondrial metabolic flexibility, as suggested by the reduction in carbohydrate over fatty acid oxidation capacity observed in granulosa cells from obese when compared to normal-weight mares. Few studies have quantified acylcarnitine species in the follicular fluid of women^38,60^, and no reports are available for mares. Concentrations of all the reported acylcarnitine species and free L-carnitine seem to be much greater (200–500 times) in follicular fluid from women than mares, but the proportions of short-, mid- and long-chain species are similar. This can be, in part, a reflection of the difference in preovulatory follicular sizes, which is 2.1 times larger in mares than women^61^. Interestingly C16:C3, which in humans is inversely related to complete oxidation of long-chain fatty acids^38^, is much lower in mares than women. This may indicate a greater participation of lipid metabolism in the follicular environment from mares in comparison to women.

In accordance with a previous report by our group^15^, the insulin concentration in follicular fluid was greater for OB than NW. In women, follicular hyperinsulinemia promotes excessive androgen production and contributes to development of polycystic ovarian syndrome^62^ and anovulation associated with increased follicular LH sensitivity and LH secretion^63^. Few equine studies have quantified follicular insulin concentrations, but obesity and increased circulating insulin are associated with prolonged estrous cycle durations and anovulation in mares^14^. Although further investigations are needed to identify specific mechanisms, obesity-associated hyperinsulinemia seems to promote similar disruptions in ovarian activity for mares and women. The extent that follicular hyperinsulinemia directly affects the developing oocyte in the obese female is not known, as its effects are difficult to isolate from other obesity-induced alterations such as hyperlipidemia and oxidative stress. Nevertheless, bovine oocyte exposure to insulin during in vitro maturation impairs development and alters embryonic organization^64^.

Excessive accumulation of lipids in cells, promoted by obesity, leads to metabolic and mitochondrial overload, increased ROS production, and cellular oxidative stress^65^. Accordingly, we observed several mitochondrial metabolic disturbances in granulosa cells from obese mares, namely excessive ROS production under basal and stimulated conditions, greater mitochondrial damage, and a trend for impaired OXPHOS efficiency especially when oxidizing pyruvate. Additionally, in obese mares, granulosa cell capacity to oxidize pyruvate when compared to fatty acids was reduced, which may be associated with limited l-carnitine availability as discussed above. Interestingly, obesity also increased the expression of complexes I and III relative to the other complexes in the granulosa cell mitochondrial respiratory chain, possibly reflecting a stoichiometric shift in electron transport kinetics. The metabolic consequence of these shifts are unclear, but complexes I and III are the primary sites of ROS production in the mitochondrial respiratory chain^66^, perhaps favoring the greater mitochondrial ROS release observed from OB granulosa cells.

Oocyte development in a lipotoxic environment is thought to be one of the main negative effects of maternal obesity on fertility, as it promotes direct impairment of oocyte quality because of lipid accumulation and metabolic and oxidative stress^67^. In the present study, oocyte aerobic and anaerobic metabolism were significantly higher in OB than in NW mares, suggesting increased oocyte metabolic activity associated with obesity. While energy production is essential for oocyte function, excess oxygen consumption could result in an increase in harmful byproducts, such as ROS, which has been shown to be detrimental to oocyte viability in obese mice^39^. To the best of our knowledge, no previous studies have described altered oocyte metabolism promoted by maternal obesity in mares, but a similar finding was recently reported in women. Oocytes from overweight women, assayed for aerobic metabolism through the same methodology utilized in this study, have higher OCR when compared to oocytes from normal-weight women^68^. In rodent models, diet-induced obesity increases oocyte mitochondrial membrane potential, mitochondrial damage, oxidative and endoplasmic reticulum stress^39,69–71^. Although oocyte ROS formation was not directly assayed in the present study, an excess metabolic rate in oocytes from obese mares could lead to oxidative stress and ultimately deleterious effects on oocyte quality.

Diet supplementation is a feasible method to influence oocyte quality in vivo. Recently, our group tested the potential of dietary supplementation to alter the follicular environment and improve oocyte quality in old mares^30^. The dietary supplement formulation utilized in this study included many of the same components as in our previous study (trace minerals, vitamins, pre- and pro-biotics, omega-3 fatty acids, natural antioxidants, among others), providing a synergistic blend of complex nutrients to improve cell metabolic function. Ingredients which specifically target obesity-induced metabolic disturbances, such as antioxidants (d-alpha-tocopherol and pterostilbene), L-carnitine, and chromium, have also been demonstrated to be metabolically beneficial for obese mice and humans^32–36,72^. Herein, we reported major positive effects of dietary intervention in improving cell metabolic function and lessening many of the negative effects of mare obesity in the ovarian follicle and oocyte. We also observed systemic effects of the dietary intervention, with improved insulin regulation and mitochondrial function of muscle cells (Fresa et al., unpublished data). While it is tempting to study individual nutrients or dietary components, the complex and synergistic mechanisms associated with metabolic health and cellular metabolic function guided our decisions to provide a complex blend of compounds to support cell metabolism and to demonstrate that nutritional interventions can be used to mitigate obesity-associated cellular alterations.

Results from the current study demonstrate that dietary nutrient supplementation fed to obese mares promoted major metabolic improvements in granulosa cells. In comparison to OB, OBD granulosa cells showed reduced ROS production and increased capacity to oxidize pyruvate versus fatty acids, with values comparable to NW. Potentially, the dietary supplement improved metabolic efficiency by increasing L-carnitine availability, which favors mitochondrial metabolic flexibility^32^. Interestingly, lowered expression of glutathione peroxidase 1 (GPX1) and superoxide dismutase (SOD2) was observed in OBD, possibly due to lower mitochondrial ROS production when obese mares were fed the dietary nutrient supplementation. Mitigation of granulosa cell ROS release with dietary nutrient supplementation, as observed in the present study, may help preserve oocyte quality with maternal obesity. Other studies investigating the effectiveness of multi-ingredient supplements, including antioxidants, vitamins and phytonutrients to reduce the negative effects of obesity in mice, have demonstrated improved reproductive outcomes, lower ovarian inflammation, and less atretic follicles^73^.

Concentrations of free L-carnitine and several acylcarnitine species were higher in follicular fluid from OBD than the other groups, indicating that L-carnitine supplemented in the diet reached the follicular environment. This could have improved the efficiency of mitochondrial metabolism in granulosa cells as suggested by our respirometry data and the lower ratio in OBD than OB of C16:C3 acylcarnitines, which in humans is indicative of more complete oxidation of long-chain fatty acids^38^. By improving mitochondrial metabolic efficiency, follicular L-carnitine could have contributed to a reduction in ROS production by granulosa cells; however, L-carnitine also has direct antioxidant actions that can potentiate this effect^74^. Taken together, these results support the conclusion that targeted supplementation combining specific levels of vitamins, trace minerals, amino acids, L-carnitine, antioxidants, omega-3 fatty acids, prebiotics and postbiotics can at least partially mitigate impairments in mitochondrial metabolism within granulosa cells in the ovarian follicles of obese mares.

Cumulus cell lipid profiles of obese mares were normalized with diet supplementation, which may contribute positively to oocyte quality. In women, there is a negative correlation between lipid content of granulosa and cumulus cells and pregnancy success after ART procedures^75^. Although cumulus cell metabolism was not directly evaluated in this study, these cells are derived from and in direct contact to mural granulosa cells^4^. Metabolism of granulosa, cumulus cells, and oocytes are linked and regulated through bi-directional communication among the cell types^76^. Thus, reduced and normalized lipids in cumulus cells from OBD may also be reflective of improved mitochondrial metabolism, as observed in the associated granulosa cells and oocytes.

Because mares in the OB group were fed grain additives to achieve obesity, we are unable to isolate potential negative effects of the grains from the effects of obesity on cellular metabolism. In terms of fetal development, both obesity and grain supplementation have negative effects that have been demonstrated in mares^17,77,78^. Starch-rich diets adversely affect oocyte quality in cows^79,80^ and may negatively affect fertility of women^81^. Nevertheless, grain additives are commonly included in the modern equine diet and predispose to obesity and metabolic diseases^82^. We are also unable to differentiate between individual and synergistic effects of the OBD dietary supplementation components. However, the beneficial effects of the supplements on cells of the ovarian follicles from obese mares demonstrated the potential of dietary interventions as an applicable therapy for obese mares and women facing reproductive challenges. OBD mares were also fed grain additives, thus any potential negative effects of grain consumption on the follicular environment were overcome by the diet supplementation components. Correction of oocyte metabolic dysfunction promoted by maternal obesity may not only contribute to improved fertility outcomes but may also aid in limiting developmental programming of metabolic disturbances in offspring.

In conclusion, in the present study, maternal obesity influenced multiple aspects of the ovarian follicular environment in mares and short-term diet interventions were able to normalize obesity-induced metabolic changes in the follicle. Further studies are needed to elucidate the translational aspects of our findings and potential effects of individual dietary supplementation ingredients.

## Methods

### Experimental design and mare feeding regimens

Mare procedures were approved by Colorado State University’s Institutional Animal Care and Use Committee. Nonlactating, light-horse mares (n = 20, 11–22 years) were matched by age and divided into three groups considering body condition scores (BCS, 1–9)^83^ upon start of the study. Mares in the Normal Weight group (NW, n = 6, mean age 17.8 ± 1.8 years) had a BCS of 5–6 at the beginning of the study. Mares included in the overweight groups were not all initially considered obese (BCS 7–9)^83^ and ranged in BCS from 6 to 8 when assigned to obese groups: Obese (OB, n = 7, mean age 18.6 ± 1.5 years); and Obese Diet Supplemented (OBD, n = 7, mean age 17.7 ± 1.4 years). Groups were housed in similar and adjacent dry lots, providing ample area for self-initiated activities; mares had no additional exercise. Hay was fed in large feeders which allowed access to all mares. NW mares were fed grass/alfalfa mix hay at approximately 2% of body weight daily and 57 g daily of a commercial vitamin and mineral forage balancer (Purina^®^ Free Balance^®^ 12:12, Purina Animal Nutrition, Gray Summit, MO, USA). OB and OBD mares were fed grass/alfalfa hay ad libitum and, twice daily, 28.5 g of the forage balancer, 0.75 kg of whole oats and 0.75 kg of cracked corn to increase their daily caloric intake and ensure achievement of obesity by sample collections. OBD mares also received targeted supplementation designed to support equine metabolic and gastrointestinal health and formulated to support cellular metabolism (187 g daily, divided into two feedings), including vitamins, trace minerals, amino acids, L-carnitine, antioxidants, omega-3 fatty acids, prebiotics and probiotics (Platinum Performance Inc., Buellton, CA, USA). Mares were monitored for body weight and multiple indicators of adiposity, including BCS (1–9)^83^, percentage of body fat (calculated from the equation: 2.47 + 5.47 * tailhead fat in cm)^84^, and cresty neck score (0–5)^85^ every 2 weeks. Tailhead fat thickness was measured with a 10 MHz, linear-array transducer positioned approximately 7.6 cm cranial and 5 cm lateral from the tailhead; fat thickness in this area has the strongest correlation to BCS^86^. Mares were provided group-specific feeding regimes for 6–10 weeks before follicular sample collections in August and September.

### Sample collection from preovulatory follicles

Follicular maturation was induced during the follicular phase and when the dominant follicle was ≥ 35 mm in diameter and uterine endometrial edema, consistent with estrus, was observed by ultrasonography. Administration of a GnRH analog, histrelin in an aqueous base (0.5 mg, IM; Doc Lane, Lexington, KY, USA), was used to induce follicle and oocyte maturation to approximately the metaphase I stage of oocyte development prior to sample collection. Follicular fluid, cumulus-oocyte complexes and granulosa cells were collected by transvaginal, ultrasound-guided follicular aspirations of dominant follicles at 20 ± 2 h after induction, as previously described^87^. Briefly, upon puncture of the follicle, approximately 10 ml of follicular fluid was aspirated from the center of the follicular antrum to minimize cellular contamination; the follicle was then rinsed with a physiological salt solution as the follicular contents were aspirated into a sterile bottle. Follicular fluid samples were aliquoted and stored at − 80 °C until assays. The remaining aspirate was searched under a stereoscope to recover the cumulus oocyte complex, which was identified by the large cumulus cell complex surrounding the oocyte. The follicular aspirates typically contained abundant granulosa cells, which were aspirated from the follicular wall and imaged as large, granular sheets of cells under a stereomicroscope. Sections of granulosa cells were manually pulled from the aspirate sample into a 15 mL conical tube, rinsed in flush solution (Vigro Complete Flush Solution, Vetoquinol, Fort Worth, TX, USA), vortexed and centrifuged at 750 × *g* for 5 min. The cell pellet (approximately 0.5 mL) was resuspended for 1 min in red blood cell lysis buffer at 37 °C, before dilution in 3 mL holding medium (TCM 199 with Hank’s salts and 10% fetal bovine serum), and was centrifuged at 750 × *g* for 5 min. The pellet was then resuspended in PBS, centrifuged at same previous settings, and either resuspended in 1 mL of flush solution and held at 4 °C until high-resolution respirometry assays, or snap frozen and stored at − 80 °C for later protein and RNA isolation. For lipid composition analyses, recovered cumulus oocytes complexes were evaluated and trimmed of any attached granulosa cells; the cumulus oocyte complexes were then placed in hyaluronidase (80 U/mL). A section of cumulus cells was manually trimmed and separated; the oocyte was then denuded of remaining cumulus cells by sequential pipetting with a stripper pipette. Individual oocytes and their respective cumulus cells were then separately rinsed and fixed in 100 μL of 50% methanol solution, snap-frozen in liquid nitrogen, and stored at − 80 °C until mass spectrometry analyses.

For metabolic function assays, additional cumulus-oocyte complexes were collected as described above and incubated in medium (TCM199 with Earle’s salts with 10% fetal bovine serum and 25 μg/mL of gentamicin) at 38.2 °C in 5% CO_2_ and air for 22 ± 2 h for the completion of oocyte maturation to the metaphase II stage. Matured oocytes were stripped of cumulus cells to confirm extrusion of the first polar body. For electrochemical measurements, single oocytes were held in a MOPS-buffered medium (G-MOPS™, Vitrolife, Englewood, CO, USA) at 4 °C until microsensor assays.

### Granulosa cell high-resolution respirometry

Intact and live granulosa cells were resuspended in 250 μL of mitochondrial respiration medium (MiR05) containing (in mM) 0.5 EGTA, 3 MgCl_2_ hexahydrate, 60 lactobionic acid, 20 taurine, 10 KH_2_PO4, 20 HEPES, 110 sucrose, and 0.1% BSA, pH 7.1 with KOH, then added to a 2-mL chamber in an Oxygraph-2 k high-resolution respirometer (Oroboros Instruments, Innsbruck, Austria) containing room air-saturated oxygen (~ 160 µM) in MiR05 maintained at 37 °C while stirring at 750 rpm. Results were normalized to the protein concentration of the granulosa cell sample pelleted at 10,000 × g for 10 min following each assay. Basal OCR and ROS release of intact cells was measured prior to permeabilization of cell membranes with digitonin (10 µg/mL) to provide mitochondrial access to cell-impermeable substrates. Mitochondrial oxidative phosphorylation (OXPHOS)-linked OCR was stimulated in permeabilized cells by the addition of 1 mM malate + 5 mM pyruvate or 1 mM malate + 0.05 mM palmitoylcarnitine in the presence of 2.5 mM adenosine diphosphate (ADP) to assess carbohydrate- and fatty acid-linked OXPHOS capacities, respectively. Maximal OXPHOS capacity was then measured following the addition of 10 mM succinate (fully saturating electron input through complex II), followed by the addition of 10 µM cytochrome *c* to assess mitochondrial membrane damage. Cytochrome *c* is not permeable to the outer mitochondrial membrane, and thus stimulates OCR (by donating electrons directly to cytochrome oxidase) in direct proportion to the extent of mitochondrial membrane damage present in the sample^88^. The rate of reactive oxygen species (ROS) release from samples was measured simultaneously with OCR in the OXPHOS-linked state by monitoring the development of resorufin fluorescence produced by the interaction of hydrogen peroxide released by the sample with 10 μM Amplex Red in the presence of horseradish peroxidase (1 U/mL) as previously described^89^. ROS data are presented as rate of release per second and as a proportion of concomitant OCR.

### Granulosa cell gene expression

Extractions of mRNA from frozen granulosa cell pellets were conducted using a TRIzol RNA isolation protocol. cDNA samples derived from 1000 ng of RNA and synthesized using Platinum™ PCR SuperMix. Gene expression was determined using quantitative polymerase chain reaction (qPCR) using SYBR Green (LightCycler 480 SYBR Green Master, Roche Diagnostics, Indianapolis, IN) (see Appendix A.1 for the detailed method). Quantification of mRNA transcripts from each gene of interest was normalized to a housekeeping gene (*GAPDH*). The relative expression of each gene was calculated by the delta delta CT method^90^. Target genes were specific to pathways of interest. Primer details are listed in Supplementary Table 3.

### Granulosa cell protein isolation and expression

Frozen granulosa cell pellets were thawed and resuspended in M-PER™ Mammalian Protein Extraction Reagent lysis buffer containing Halt™ Protease and Phosphatase Inhibitor Single-Use Cocktail (100X). Homogenates were sonicated (Branson 250 Digital Sonifier Ultrasonic Cell Disruptor, Branson Ultrasonics Corporation), and centrifuged at 10,000 × *g* for 10 min at 4 °C. Supernatants were transferred to new microcentrifuge tubes and stored at -80 °C. Each sample was analyzed for total protein using Bicinchoninic acid (BCA) assay before immunoblotting.

Samples (40 μL) containing 30 μg of granulosa cell proteins, Bolt™ Sample Reducing Agent, and 2 × Laemmli Sample Buffer were added to 4–12% Bis–Tris polyacrylamide gels and electrophoresed for 1 h at 150 V. Protein was then transferred to polyvinylidene difluoride membranes, blocked in 5% non-fat milk for 1 h, then rocked overnight in 5% non-fat milk containing 1:1000 primary antibody at 4 °C. Antibodies for mitochondrial electron transport system complexes (Total OXPHOS, MS604300, Abcam, Boston, MA), SOD1 and SOD2 (SOD-101 and SOD-111, Stressgen Biotechnologies Corp., Victoria, British Columbia, Canada) and GPX1 (PA526323, Invitrogen, Thermo Fisher Scientific, Waltham, MA) were used. Membranes were washed for 5 min 3 times in Tris-buffered saline + Tween (TBST; 20 mm Tris-base, 150 mm NaCl, pH 7.4) prior to addition of 5% non-fat milk containing 1:3000 secondary antibody. Membranes were rocked for 1 h, washed in TBST, and incubated with chemiluminescence (SuperSignal™ West Dura Extended Duration Substrate, Thermo Fisher) at room temperature for 1 min prior to imaging. After imaging, membranes were stained for total protein using Amido Black^91^ (Thermo Fisher) and imaged. Target protein concentrations were quantified with densitometric analysis and standardized to Amido Black. Uncropped membrane pictures are provided in Supplementary Information 2.

### Determination of follicular fluid insulin, lipid and acylcarnitine concentrations

Triglyceride concentrations in follicular fluid samples were determined using a colorimetric assay kit (Cayman Chemical, Ann Harbor, MI, USA) according to kit instructions. The 96-well, non-treated microplate was read at 540-nm absorbance on a Synergy 2 microplate reader (Biotek, Agilent, Santa Clara, CA, USA). All samples were assayed on a single plate. The intra-assay coefficient of variation was 1.35%, and the minimal detectable concentration was 1 mg/dL. Concentrations of non-esterified free fatty acids and insulin in follicular fluid were determined by a reference laboratory (Clinical Pathology Laboratory, Cornell University Animal Health Diagnostic Center, Ithaca, NY). Analyses of follicular fluid acylcarnitine profiles were conducted by a reference laboratory (University of Colorado Anschutz Medical Campus School of Medicine Metabolomics Core, Aurora, CO, USA) as previously described^92^ (see Appendix A.2 for the detailed method).

### Cumulus cell and oocyte lipidomics analyses by liquid chromatography coupled to mass spectrometry

Samples were lyophilized to remove water. 500 μL of cold 100% methanol spiked with 0.33 μg/mL cholesterol 2,2,3,4,4,6-D6, 97–98% -d6 was added to each sample. Three blank samples were included in randomized order. Samples were briefly vortexed, sonicated in a cold bath for 5 min, then shaken at 4 °C for 30 min, briefly sonicated, and centrifuged at 15,000 × g for 10 min at 4 °C. Samples (470 μL) were recovered and dried under nitrogen. Oocyte extracts were resuspended in 60 μL 2:1 methanol/toluene, and cumulus cell extracts were resuspended in 20 μL 2:1 methanol/toluene. From each oocyte sample, 20 μL were collected and pooled for an oocyte quality control (QC) sample. From each cumulus cell sample, 5 μL were collected for a cumulus cell QC. The samples were then transferred to inserts for direct LCMS injection. One microliter of extract was injected onto a ACQUITY UPLC system (Waters, Milford, MA, USA) in randomized order with a pooled QC injection after every six samples, as previously described by this laboratory^30^. XCMS (version 3.16.1) in R (version 4.1.2) was used for feature finding, retention time alignment, correspondence analysis, and peak filling^93,94^. RAMClustR (version 1.2.2) in R (version 4.0.5) was used to normalize, filter, and group features into spectra^95^, (see Appendix A.3 for the detailed method).

### Oocyte metabolic function assays (OCR and ECAR)

Assays of single oocyte metabolic function were performed using a microchamber with electrochemical-based oxygen and pH sensors. Fabrication of the electrochemical sensor chips and hardware in the device used in this study were previously reported^27,96^. The microchamber was filled with 180 μL of MOPS-buffered medium (G-MOPS™) and placed inside an incubator at 38.5 °C^96^. Approximately 15 min before the assay, each denuded oocyte was warmed to 38.5 °C and pipetted onto the working electrode of the oxygen sensor, after oxygen and pH sensors had reached a steady baseline state. OCR and ECAR were measured, respectively, with amperometric and potentiometric sensors^27^.

### Statistical analyses

For most analyses, a single sample from each mare (NW, n = 6; OB, n = 7, and OBD, n = 6) was included. However, western blots were performed on granulosa cells from five individual mares per group. Lipidomics of oocytes and cumulus cells included five mares from NW, as one mare in NW was consistently considered an outlier based on ROUT test. GraphPad Prism 9.3.1 was used. Continuous data were analyzed for normality by Shapiro–Wilk tests. Repeated body weight, BCS, percentage body fat, and cresty neck score measures were analyzed within and among groups by two-way ANOVA with post-hoc Tukey’s multiple comparison tests. One-way ANOVA with post-hoc Tukey’s multiple comparison tests were used to compare normally distributed data sets. Kruskal–Wallis tests, followed by Dunn’s multiple comparison tests were used for data that failed normality. Values of P < 0.05 were considered significant, and P ≤ 0.1 was considered tending toward significance. Results are presented as mean ± SEM.

### Supplementary Information

Supplementary Information 1.

Supplementary Information 2.

Supplementary Information 3.

## Data Availability

The data generated during and/or analyzed in the current study are available from the corresponding authors upon reasonable request.
