# Supplementary material for: Follicular metabolic alterations are associated with obesity in mares and can be mitigated by dietary supplementation
Source: Sci Rep. 2024 Mar 30;14:7571. doi: 10.1038/s41598-024-58323-0 (PMC10981747; doi:10.1038/s41598-024-58323-0)
Supplement: Supplementary file 1 — Supplementary Information 1. [file 41598_2024_58323_MOESM1_ESM.docx]

**Follicular metabolic alterations are associated with obesity in mares and can be mitigated by dietary supplementation**

Giovana D Catandi^A,B^, Kyle Fresa^A,B^, Ming-Hao Cheng^C^, Luke Whitcomb^B^, Corey D Broeckling^D^, Thomas W Chen^C,E^, Adam J Chicco^B^, Elaine M Carnevale^A,B,*^

^A^Equine Reproduction Laboratory, Department of Biomedical Sciences, Colorado State University, 3101 Rampart Road, Fort Collins, CO 80521, USA.

^B^Department of Biomedical Sciences, Colorado State University, Fort Collins, CO 80523, USA.

^C^Department of Electrical and Computer Engineering, Colorado State University, Fort Collins, CO 8523, USA.

^D^Proteomics and Metabolomics Facility, Colorado State University, Fort Collins, CO 80523, USA.

^E^School of Biomedical Engineering, Colorado State University, Fort Collins, CO 80523, USA.

^*^Corresponding author. Email: [elaine.carnevale@colostate.edu](mailto:elaine.carnevale@colostate.edu)

**Appendix: Detailed methods used for assessment of follicular cells and oocytes**

1. **Granulosa cell gene expression**

Granulosa cells were thawed on ice and homogenized via vigorous pipetting in 1 mL of TRI Reagent^®^. Samples were held at room temperature for 10 min and then centrifuged at 12000 x g for 10 min at 4^o^C to remove cell debris. The clear supernatant was transferred to a new microtube. A solution of 100 μL of 1-bromo-3-chloropropane was added to the sample, which was shook vigorously and centrifuged at 12000 x g for 15 min at 4^o^C to form an RNA-containing aqueous layer. The clear aqueous layer was removed, and 500 μL of 2-propanol was added and centrifuged. Visible RNA pellets were washed in 1 mL of 75% ethanol, centrifuged at 7500 x g for 5 min at 4^o^C, and resuspended in 50 μL of water. RNA concentration and purity were analyzed using a NanoDrop™ spectrophotometer.

Complementary DNA samples were derived from 1000 ng of RNA and synthesized using Platinum^TM^ PCR SuperMix. Gene expression was determined using quantitative polymerase chain reaction (qPCR) using SYBR Green (LightCycler 480 SYBR Green Master, Roche Diagnostics, Indianapolis, IN). Samples were run in triplicate in a 10-mL reaction using a LightCycler 480II (Roche Diagnostics) and the program: 95^o^C for 10 min, followed by 45 cycles at 95^o^C for 30 s, 60^o^C for 60 s, and 72^o^C for 60 s. Quantification of mRNA transcripts from each gene of interest was normalized to a housekeeping gene (*GAPDH*). The relative expression of each gene was calculated by the delta delta CT method^1^. Target genes were specific to pathways of interest: carnitine-associated mitochondrial enzymes (carnitine acetyltransferase, *CRAT*; carnitine palmitoyltransferase IB, *CPT1B*), regulation of pyruvate dehydrogenase (pyruvate dehydrogenase kinase 4, *PDK4*; pyruvate dehydrogenase phosphatase 1, *PDP1*), anaerobic glycolysis (lactate dehydrogenase A, *LDHA*), fatty acid synthesis (fatty acid synthase, *FASN*), mitochondrial folate one-carbon metabolism (methylenetetrahydrofolate dehydrogenase 2, *MTHFD2*), lipogenesis (sterol regulatory element-binding protein1, *SREBF1*), cholesterol homeostasis (sterol regulatory element-binding protein 2, *SREBF2*), granulosa cell proliferation (cyclin D2, *CCND2*), and steroidogenesis (luteinizing hormone receptor, *LHCGR*; steroidogenic acute regulatory protein, *STAR*; cholesterol side-chain cleavage enzyme, *CYP11A1*; aromatase, *CYP19A1*). Specific primers for each gene were designed using Primer3 (<http://bioinfo.ut.ee/primer3-0.4.0/>) and validated by RT-PCR. Primer details are listed in Supplementary Table 3.

1. **Follicular fluid acylcarnitine concentrations**

Follicular fluid (20 µL) was aliquoted, before the addition of 180 uL of an internal standard solution compromised of known concentrations of acylcarnitine species in methanol. Samples were briefly vortexed and incubated at 20^o^C for 15 min. Samples were subsequently centrifuged at 12700 RPM for 10 min at 4^o^C; 100 µL of supernatant were transferred to a new tube, and 100 µL of 10 mM ammonium acetate were added to each sample. A 10-uL allotment of each sample was injected and analyzed via a Thermo Vanquisher Liquid Chromatography system coupled to a Q-Exactive™ Quadrupole-Orbitrap™ Mass Spectrometer as described in detail^2^. Data analysis was done using Maven software^2^.

1. **Cumulus cell and oocyte lipidomic LC-MS data extraction**

XCMS (version 3.16.1) in R (version 4.1.2) was used for feature finding, retention time alignment, correspondence analysis, and peak filling^3,4^. RAMClustR (version 1.2.2) in R (version 4.0.5) was used to normalize, filter, and group features into spectra^5^, (see Appendix A.2 for the detailed method). XCMS output data was transferred to a ramclustR object using the rc.get.xcms.data function. Feature data was extracted using the xcms featureValues function. Features which failed to demonstrate signal intensity of at least 3-fold greater in samples than in prep blanks were removed from the feature dataset. Molecular weight was inferred from MS1 spectra using the do.findmain function^6^, which calls the interpretMSSpectrum package^7^. MSFinder was used for spectral matching^8^, and both MSFinder and Sirius for structure inference^9^. Annotations were assigned using the RAMClustR annotate function based only on MSFinder output – Sirius output is available to complement the annotation efforts. Compounds were assigned to chemical ontogenies using the ClassyFire API^10^. Pubchem data was retrieved using the Pubchem API.

1. Pfaffl, M. W. A new mathematical model for relative quantification in real-time RT-PCR. *Nucleic Acids Research* **29**, 45e–445 (2001).

2. Reisz, J. A., Zheng, C., D’Alessandro, A. & Nemkov, T. Untargeted and Semi-targeted Lipid Analysis of Biological Samples Using Mass Spectrometry-Based Metabolomics. in *High-Throughput Metabolomics: Methods and Protocols* (ed. D’Alessandro, A.) 121–135 (Springer New York, 2019). doi:10.1007/978-1-4939-9236-2_8.

3. Smith, C. A., Want, E. J., O’Maille, G., Abagyan, R. & Siuzdak, G. XCMS:  Processing Mass Spectrometry Data for Metabolite Profiling Using Nonlinear Peak Alignment, Matching, and Identification. *Anal. Chem.* **78**, 779–787 (2006).

4. Tautenhahn, R., Böttcher, C. & Neumann, S. Highly sensitive feature detection for high resolution LC/MS. *BMC Bioinformatics* **9**, 504 (2008).

5. Broeckling, C. D., Afsar, F. A., Neumann, S., Ben-Hur, A. & Prenni, J. E. RAMClust: A Novel Feature Clustering Method Enables Spectral-Matching-Based Annotation for Metabolomics Data. *Anal. Chem.* **86**, 6812–6817 (2014).

6. Broeckling, C. D. *et al.* Enabling Efficient and Confident Annotation of LC−MS Metabolomics Data through MS1 Spectrum and Time Prediction. *Anal. Chem.* **88**, 9226–9234 (2016).

7. Jaeger, C., Méret, M., Schmitt, C. A. & Lisec, J. Compound annotation in liquid chromatography/high-resolution mass spectrometry based metabolomics: robust adduct ion determination as a prerequisite to structure prediction in electrospray ionization mass spectra. *Rapid Commun Mass Spectrom* **31**, 1261–1266 (2017).

8. Tsugawa, H. *et al.* Hydrogen Rearrangement Rules: Computational MS/MS Fragmentation and Structure Elucidation Using MS-FINDER Software. *Anal. Chem.* **88**, 7946–7958 (2016).

9. Dührkop, K., Shen, H., Meusel, M., Rousu, J. & Böcker, S. Searching molecular structure databases with tandem mass spectra using CSI:FingerID. *Proc. Natl. Acad. Sci. U.S.A.* **112**, 12580–12585 (2015).

10. Djoumbou Feunang, Y. *et al.* ClassyFire: automated chemical classification with a comprehensive, computable taxonomy. *J Cheminform* **8**, 61 (2016).
