# Supplementary material for: Follicular metabolic alterations are associated with obesity in mares and can be mitigated by dietary supplementation
Source: Sci Rep. 2024 Mar 30;14:7571. doi: 10.1038/s41598-024-58323-0 (PMC10981747; doi:10.1038/s41598-024-58323-0)
Supplement: Supplementary file 2 — Supplementary Information 2. [file 41598_2024_58323_MOESM2_ESM.docx]

**Supplementary Figure S1. Morphometric measurements of mares.** Morphometric measurements from normal weight (NW, n=6), obese (OB, n=7) and obese diet supplemented (ODB, n=7) mares were performed at 2-week intervals: (**a**) body weight in kg, (**b**) body condition score (scored from 1, emaciated to 9, extreme obesity^1^), (**c**) percentage of body fat^2^, and (**d**) cresty neck score (0, none to 5, large crest dropped to one side^3^). Graphs represent mean ± SEM. Asterisks denote differences in a given week between NW and obese groups (OB and OBD) using two-way ANOVA (P<0.05).

**Supplementary Figure S2. Effects of mare obesity and diet supplementation on granulosa cell gene expression.** Expression of genes of interest in granulosa cells obtained from preovulatory follicles of normal-weight (NW, n=6), obese (OB, n=7) and obese diet supplemented (OBD, n=6) mares after ≥ 6 weeks of supplementation. Data were normalized to expression of a housekeeping gene (*GAPDH*: glyceraldehyde 3-phosphate dehydrogenase) and are presented as fold changes relative to the control group (NW): (**a**) *CRAT*: carnitine acetyltransferase, (**b**) *CPT1B:* carnitine palmitoyltransferase IB, (**c**) *PDK4:* pyruvate dehydrogenase kinase 4, (**d**) *PDP1:* pyruvate dehydrogenase, (**e**) *LDHA:* lactate dehydrogenase A, (**f**) *FASN:* fatty acid synthase, (**g**) *MTHFD2:* methylenetetrahydrofolate dehydrogenase 2, (**h**) *SREBF1:* sterol regulatory element-binding protein1, (**i**) *SREBF2:* sterol regulatory element-binding protein 2, (**j**) *CCND2:* cyclin D2, (**k**) *LHCGR:* luteinizing hormone receptor, (**l**) *STAR:* steroidogenic acute regulatory protein, (**m**) *CYP11A1:* cholesterol side-chain cleavage enzyme, and (**n**) *CYP19A1:* aromatase. Graphs represent mean ± SEM. Different superscripts indicate difference (^ab^, P<0.05) between groups using one-way ANOVA with post-hoc Tukey’s multiple comparison tests, or Kruskal-Wallis tests, followed by Dunn’s multiple comparison tests.

**Supplementary Table 1.** Abundance of lipids in cumulus cells that differed between groups (NW; n=5, OB, n=7; OBD, n=6). Results are presented as mean ± SEM. Different superscripts within the same row indicate difference (^ab^, P < 0.05) or tendency for difference (^cd^, P < 0.1) between groups using one-way ANOVA with post-hoc Tukey’s multiple comparison tests.

| Lipid species | NW | OB | OBD | P value  (NW x OB) | P value  (NW x OBD) | | P value  (OB x OBD) |  |
| --- | --- | --- | --- | --- | --- | --- | --- | --- |
| TG(62:5) | 0.22 x 10^5^ ± 0.11^c^ | 1.31 x 10^5^ ± 0.40^d^ | 0.46 x 10^5^ ± 0.12^cd^ | 0.06 | 0.8 | 0.2 | | |
| TG(58:4) | 1.57 x 10^5^ ± 0.67^c^ | 5.70 x 10^5^ ± 1.31^d^ | 3.35 x 10^5^ ± 0.70^cd^ | 0.08 | 0.5 | 0.5 | | |
| TG(60:3) | 0.21 x 10^5^ ± 0.09^ac^ | 1.25 x 10^5^ ± 0.33^bd^ | 0.36 x 10^5^ ± 0.12^c^ | 0.03 | 0.9 | 0.09 | | |
| TG(60:4) | 0.32 x 10^5^ ± 0.15^a^ | 1.71 x 10^5^ ± 0.48^b^ | 0.65 x 10^5^ ± 0.18^ab^ | 0.05 | 0.8 | 0.2 | | |
| TG(68:7) | 0.97 x 10^4^ ± 0.42^c^ | 6.40 x 10^4^ ± 2.07^d^ | 2.15 x 10^4^ ± 0.32^cd^ | 0.06 | 0.9 | 0.2 | | |
| TG(54:2) | 1.30 x 10^6^ ± 0.47^c^ | 3.74 x 10^6^ ± 0.77^d^ | 2.17 x 10^6^ ± 0.26^cd^ | 0.08 | 0.6 | 0.4 | | |
| TG(54:1) | 1.79 x 10^5^ ± 0.58^a^ | 5.90 x 10^5^ ± 1.01^b^ | 2.94 x 10^5^ ± 0.45^ab^ | 0.02 | 0.6 | 0.1 | | |
| TG(56:3) | 0.44 x 10^6^ ± 0.18^c^ | 1.33 x 10^6^ ± 0.27^d^ | 0.83 x 10^6^ ± 0.15^cd^ | 0.09 | 0.5 | 0.5 | | |
| TG(58:3) | 0.73 x 10^5^ ± 0.33^c^ | 3.27 x 10^5^ ± 0.88^d^ | 1.44 x 10^5^ ± 0.37^cd^ | 0.07 | 0.7 | 0.3 | | |
| TG(60:2) | 1.20 x 10^4^ ± 0.32^a^ | 5.22 x 10^4^ ± 1.19^b^ | 1.37 x 10^4^ ± 0.34^a^ | 0.02 | 0.97 | 0.03 | | |
| TG(50:1) | 0.92 x 10^6^ ± 0.19^c^ | 1.72 x 10^6^ ± 0.22^d^ | 1.17 x 10^6^ ± 0.08^cd^ | 0.08 | 0.6 | 0.4 | | |
| TG(56:2) | 1.04 x 10^5^ ± 0.42^c^ | 3.93 x 10^5^ ± 0.88^d^ | 2.05 x 10^5^ ± 0.55^cd^ | 0.06 | 0.6 | 0.3 | | |
| TG(50:2) | 1.53 x 10^6^ ± 0.40^c^ | 3.22 x 10^6^ ± 0.45^d^ | 2.32 x 10^6^ ± 0.25^cd^ | 0.09 | 0.4 | 0.6 | | |
| TG(60:5) | 0.89 x 10^5^ ± 0.40^c^ | 4.06 x 10^5^ ± 1.17^d^ | 2.16 x 10^5^ ± 0.50^cd^ | 0.09 | 0.6 | 0.5 | | |
| TG(58:2) | 0.31 x 10^5^ ± 0.10^ac^ | 1.48 x 10^5^ ± 0.34^bd^ | 0.47 x 10^5^ ± 0.13^c^ | 0.02 | 0.9 | 0.06 | | |
| TG(62:4) | 1.40 x 10^4^ ± 0.76^a^ | 8.86 x 10^4^ ± 2.66^b^ | 2.61 x 10^4^ ± 0.70^ab^ | 0.05 | 0.9 | 0.1 | | |
| TG(54:5) | 0.40 x 10^4^ ± 0.12^a^ | 1.88 x 10^4^ ± 0.54^b^ | 0.69 x 10^4^ ± 0.07^ab^ | 0.04 | 0.8 | 0.1 | | |
| TG(48:3) | 2.94 x 10^5^ ± 0.79^c^ | 8.68 x 10^5^ ± 0.13^d^ | 5.17 x 10^5^ ± 0.76^cd^ | 0.09 | 0.7 | 0.3 | | |
| TG(52:1) | 0.99 x 10^6^ ± 0.32^c^ | 2.55 x 10^6^ ± 0.51^d^ | 1.58 x 10^6^ ± 0.18^cd^ | 0.09 | 0.6 | 0.5 | | |
| TG(52:0) | 2.55 x 10^5^ ± 0.55^a^ | 5.09 x 10^5^ ± 0.68^b^ | 3.53 x 10^5^ ± 0.16^ab^ | 0.02 | 0.4 | 0.2 | | |
| TG(64:5) | 0.62 x 10^4^ ± 0.33^a^ | 3.93 x 10^4^ ± 1.17^b^ | 1.24 x 10^4^ ± 0.34^ab^ | 0.05 | 0.8 | 0.1 | | |
| TG(56:1) | 0.38 x 10^5^ ± 0.08^a^ | 1.47 x 10^5^ ± 0.27^b^ | 0.49 x 10^5^ ± 0.07^a^ | 0.008 | 0.9 | 0.02 | | |
| TG(66:4) | 0.75 x 10^4^ ± 0.08^c^ | 1.29 x 10^4^ ± 0.21^d^ | 0.85 x 10^4^ ± 0.05^cd^ | 0.07 | 0.8 | 0.2 | | |
| TG(58:10) | 0.35 x 10^5^ ± 0.09^a^ | 1.17 x 10^5^ ± 0.16^b^ | 0.45 x 10^5^ ± 0.05^a^ | 0.005 | 0.8 | 0.02 | | |
| TG(50:3) | 1.03 x 10^4^ ± 0.14^c^ | 1.69 x 10^4^ ± 0.14^d^ | 1.15 x 10^4^ ± 0.06^cd^ | 0.08 | 0.9 | 0.2 | | |
| TG(52:4) | 0.23 x 10^4^ ± 0.09^c^ | 1.01 x 10^4^ ± 0.33^d^ | 0.32 x 10^4^ ± 0.03^cd^ | 0.09 | 0.97 | 0.1 | | |
| TG(55:3) | 1.62 x 10^3^ ± 0.23^a^ | 6.27 x 10^3^ ± 1.26^b^ | 3.32 x 10^3^ ± 0.85^ab^ | 0.05 | 0.6 | 0.3 | | |
| TG(54:4) | 2.51 x 10^3^ ± 0.40^c^ | 6.55 x 10^3^ ± 1.34^d^ | 3.74 x 10^3^ ± 0.42^cd^ | 0.06 | 0.7 | 0.2 | | |
| TG(64:4) | 0.40 x 10^4^ ± 0.19^c^ | 2.22 x 10^4^ ± 0.72^d^ | 0.76 x 10^4^ ± 0.24^cd^ | 0.07 | 0.8 | 0.2 | | |
| TG(50:1) | 1.31 x 10^4^ ± 0.48^c^ | 3.43 x 10^4^ ± 0.73^d^ | 2.22 x 10^4^ ± 0.22^cd^ | 0.09 | 0.5 | 0.5 | | |
| TG(54:3) | 6.29 x 10^4^ ± 0.79^c^ | 3.71 x 10^4^ ± 0.59^d^ | 4.74 x 10^4^ ± 0.60^cd^ | 0.09 | 0.3 | 0.8 | | |
| TG(60:11) | 3.32 x 10^3^ ± 0.58^a^ | 8.99 x 10^3^ ± 1.20^b^ | 7.17 x 10^3^ ± 1.02^ab^ | 0.05 | 0.2 | 0.8 | | |
| TG(54:5) | 2.43 x 10^3^ ± 0.43^a^ | 7.79 x 10^3^ ± 1.11^b^ | 4.25 x 10^3^ ± 0.48^ab^ | 0.03 | 0.7 | 0.2 | | |
| TG(61:4) | 0.22 x 10^4^ ± 0.10^c^ | 1.44 x 10^4^ ± 0.45^d^ | 0.43 x 10^4^ ± 0.17^cd^ | 0.06 | 0.9 | 0.1 | | |
| TG(53:1) | 0.39 x 10^4^ ± 0.06^a^ | 1.68 x 10^4^ ± 0.43^b^ | 0.46 x 10^4^ ± 0.09^a^ | 0.02 | 0.97 | 0.04 | | |
| TG(50:2) | 0.88 x 10^5^ ± 0.21^a^ | 1.77 x 10^5^ ± 0.10^b^ | 1.15 x 10^5^ ± 0.11^ab^ | 0.04 | 0.6 | 0.2 | | |
| TG(50:2) | 0.91 x 10^4^ ± 0.26^c^ | 2.31 x 10^4^ ± 0.29^d^ | 1.85 x 10^4^ ± 0.18^cd^ | 0.08 | 0.2 | 0.9 | | |
| TG(58:10) | 1.03 x 10^4^ ± 0.14^ab^ | 1.13 x 10^4^ ± 0.23^a^ | 0.50 x 10^4^ ± 0.17^b^ | 0.8 | 0.2 | 0.04 | | |
| DG(36:1) | 3.83 x 10^4^ ± 0.74^a^ | 9.73 x 10^4^ ± 1.58^b^ | 5.65 x 10^4^ ± 0.31^ab^ | 0.02 | 0.5 | 0.1 | | |
| DG(36:1) | 0.38 x 10^5^ ± 0.12^a^ | 1.23 x 10^5^ ± 0.23^b^ | 0.71 x 10^5^ ± 0.08^ab^ | 0.04 | 0.5 | 0.3 | | |
| DG(38:5) | 6.39 x 10^2^ ± 0.54^c^ | 7.26 x 10^2^ ± 0.90^cd^ | 8.47 x 10^2^ ± 0.79^d^ | 0.5 | 0.09 | 0.5 | | |
| DG(40:2) | 1.14 x 10^4^ ± 0.40^cd^ | 1.13 x 10^4^ ± 0.22^c^ | 0.46 x 10^4^ ± 0.16^d^ | 0.9 | 0.2 | 0.08 | | |
| EPA^1^ | 1.29 x 10^4^ ± 0.32^c^ | 1.03 x 10^4^ ± 0.26^cd^ | 0.46 x 10^4^ ± 0.18^d^ | 0.9 | 0.07 | 0.1 | | |
| CMPF^2^ | 5.20 x 10^5^ ± 1.10^ac^ | 4.55 x 10^5^ ± 1.21^c^ | 1.84 x 10^5^ ± 0.39^bd^ | 0.96 | 0.05 | 0.06 | | |
| PiA^3^ | 3.34 x 10^4^ ± 0.75^a^ | 1.61 x 10^4^ ± 0.22^ab^ | 0.76 x 10^4^ ± 0.16^b^ | 0.2 | 0.01 | 0.2 | | |
| PA^4^ | 2.04 x 10^4^ ± 0.43^c^ | 2.18 x 10^4^ ± 0.27^c^ | 3.01 x 10^4^ ± 0.24^d^ | 0.99 | 0.09 | 0.08 | | |
| EA^5^ | 3.77 x 10^3^ ± 0.45^a^ | 4.81 x 10^3^ ± 0.26^ab^ | 4.89 x 10^3^ ± 0.24^b^ | 0.1 | 0.05 | 0.8 | | |
| PC(38:2) | 0.34 x 10^7^ ± 0.15^c^ | 2.26 x 10^7^ ± 0.71^d^ | 0.68 x 10^7^ ± 0.07^cd^ | 0.06 | 0.9 | 0.1 | | |
| PC(38:4) | 2.70 x 10^5^ ± 0.82^cd^ | 1.75 x 10^5^ ± 0.76^c^ | 4.34 x 10^5^ ± 0.82^d^ | 0.4 | 0.5 | 0.06 | | |
| PC(36:4) | 0.72 x 10^4^ ± 0.10^ac^ | 1.70 x 10^4^ ± 0.25^bd^ | 0.85 x 10^4^ ± 0.11^c^ | 0.05 | 0.96 | 0.08 | | |
| PC(36:5) | 3.85 x 10^3^ ± 1.71^c^ | 9.98 x 10^3^ ± 1.80^d^ | 9.65 x 10^3^ ± 1.41^d^ | 0.06 | 0.06 | 0.99 | | |
| PC(32:0) | 0.62 x 10^5^ ± 0.24^c^ | 2.13 x 10^5^ ± 0.37^d^ | 2.03 x 10^5^ ± 0.40^d^ | 0.08 | 0.08 | 0.99 | | |
| PC(44:7) | 1.59 x 10^4^ ± 0.57^a^ | 3.98 x 10^4^ ± 0.59^ab^ | 4.34 x 10^4^ ± 0.66^b^ | 0.1 | 0.02 | 0.6 | | |
| PC(40:1) | 0.54 x 10^4^ ± 0.24^a^ | 2.40 x 10^4^ ± 0.68^b^ | 1.08 x 10^4^ ± 0.12^ab^ | 0.04 | 0.7 | 0.2 | | |
| PC(34:3) | 1.16 x 10^4^ ± 0.32^c^ | 3.38 x 10^4^ ± 0.78^d^ | 1.69 x 10^4^ ± 0.28^cd^ | 0.07 | 0.8 | 0.2 | | |
| PC(38:5) | 0.83 x 10^4^ ± 0.34^c^ | 3.57 x 10^4^ ± 1.07^d^ | 1.12 x 10^4^ ± 0.22^cd^ | 0.09 | 0.97 | 0.1 | | |
| LPC(14:0) | 3.90 x 10^5^ ± 0.47^ab^ | 5.72 x 10^5^ ± 0.87^a^ | 3.70 x 10^5^ ± 0.33^b^ | 0.1 | 0.9 | 0.05 | | |
| LPC(17:0) | 0.17 x 10^5^ ± 0.06^c^ | 1.02 x 10^5^ ± 0.35^d^ | 0.37 x 10^5^ ± 0.10^cd^ | 0.09 | 0.9 | 0.2 | | |
| LPC(16:0) | 1.86 x 10^5^ ± 0.04^ab^ | 2.30 x 10^5^ ± 0.32^a^ | 1.46 x 10^5^ ± 0.21^b^ | 0.3 | 0.4 | 0.02 | | |
| LPC(22:0) | 0.47 x 10^4^ ± 0.15^ac^ | 1.07 x 10^4^ ± 0.17^d^ | 1.07 x 10^4^ ± 0.14^bd^ | 0.06 | 0.03 | 0.9 | | |
| PE(40:4) | 3.84 x 10^5^ ± 1.01^ab^ | 1.86 x 10^5^ ± 0.81^a^ | 6.00 x 10^5^ ± 1.10^b^ | 0.2 | 0.3 | 0.008 | | |
| PE(40:5) | 3.95 x 10^5^ ± 0.84^cd^ | 3.45 x 10^5^ ± 0.45^c^ | 5.69 x 10^5^ ± 0.96^d^ | 0.7 | 0.4 | 0.09 | | |
| PE(29:1) | 0.81 x 10^4^ ± 0.18^a^ | 1.46 x 10^4^ ± 0.15^b^ | 1.26 x 10^4^ ± 0.17^ab^ | 0.05 | 0.2 | 0.8 | | |
| PE(38:5) | 8.16 x 10^3^ ± 0.44^ac^ | 0.73 x 10^3^ ± 0.36^bd^ | 1.48 x 10^3^ ± 0.28^d^ | 0.05 | 0.09 | 0.96 | | |
| PE(38:3) | 3.02 x 10^4^ ± 0.53^a^ | 4.70 x 10^4^ ± 1.13^ab^ | 6.49 x 10^4^ ± 0.71^b^ | 0.6 | 0.03 | 0.2 | | |
| PE(40:9) | 0.70 x 10^3^ ± 0.13^c^ | 1.17 x 10^3^ ± 0.27^cd^ | 1.34 x 10^3^ ± 0.17^d^ | 0.2 | 0.09 | 0.8 | | |
| LPE(22:5) | 2.79 x 10^3^ ± 0.63^a^ | 1.15 x 10^3^ ± 0.22^b^ | 1.73 x 10^3^ ± 0.35^ab^ | 0.02 | 0.1 | 0.6 | | |
| LPE(16:0) | 0.88 x 10^4^ ± 0.12^c^ | 2.18 x 10^4^ ± 0.57^d^ | 1.00 x 10^4^ ± 0.04^c^ | 0.06 | 0.97 | 0.09 | | |
| LPE(22:0) | 5.69 x 10^3^ ± 1.12^c^ | 4.15 x 10^3^ ± 0.85^cd^ | 2.64 x 10^3^ ± 0.44^d^ | 0.7 | 0.08 | 0.3 | | |
| PG(31:2) | 0.68 x 10^5^ ± 0.19^ac^ | 1.44 x 10^5^ ± 0.24^bd^ | 1.46 x 10^5^ ± 0.16^d^ | 0.05 | 0.08 | 0.98 | | |
| LSM(18:0) | 1.02 x 10^5^ ± 0.16^a^ | 3.16 x 10^5^ ± 0.19^b^ | 1.43 x 10^5^ ± 0.14^a^ | 0.003 | 0.96 | 0.004 | | |
| SM(34:1) | 0.76 x 10^5^ ± 0.15^a^ | 2.09 x 10^5^ ± 0.39^b^ | 1.14 x 10^5^ ± 0.07^ab^ | 0.03 | 0.7 | 0.2 | | |
| SM(42:0) | 2.46 x 10^3^ ± 0.21^ab^ | 3.35 x 10^3^ ± 0.35^a^ | 2.02 x 10^3^ ± 0.29^b^ | 0.3 | 0.4 | 0.03 | | |
| PA(44:1) | 0.17 x 10^7^ ± 0.07^c^ | 1.01 x 10^7^ ± 0.31^d^ | 0.31 x 10^7^ ± 0.03^cd^ | 0.06 | 0.9 | 0.1 | | |
| PA(40:6) | 0.58 x 10^5^ ± 0.18^a^ | 1.21 x 10^5^ ± 0.18^b^ | 1.26 x 10^5^ ± 0.14^b^ | 0.04 | 0.03 | 0.98 | | |
| PA(39:0) | 1.01 x 10^4^ ± 0.25^a^ | 6.51 x 10^4^ ± 2.07^b^ | 3.58 x 10^4^ ± 0.61^ab^ | 0.05 | 0.4 | 0.5 | | |
| PA(42:4) | 1.50 x 10^4^ ± 0.57^cd^ | 1.50 x 10^4^ ± 0.36^c^ | 2.72 x 10^4^ ± 0.45^d^ | 0.8 | 0.3 | 0.09 | | |
| PI(34:1) | 3.11 x 10^4^ ± 1.20^a^ | 6.71 x 10^4^ ± 0.94^b^ | 7.25 x 10^4^ ± 0.91^b^ | 0.05 | 0.02 | 0.8 | | |
| PI(32:0) | 3.33 x 10^4^ ± 0.73^ac^ | 3.75 x 10^4^ ± 0.36^c^ | 4.89 x 10^4^ ± 0.16^bd^ | 0.9 | 0.05 | 0.08 | | |
| PI(36:1) | 2.76 x 10^3^ ± 0.57^c^ | 3.60 x 10^3^ ± 0.51^cd^ | 4.95 x 10^3^ ± 0.67^d^ | 0.9 | 0.07 | 0.1 | | |
| PS(36:1) | 1.55 x 10^6^ ± 0.55^c^ | 4.79 x 10^6^ ± 0.96^d^ | 2.45 x 10^6^ ± 0.61^cd^ | 0.09 | 0.8 | 0.3 | | |
| PS(38:5) | 2.21 x 10^5^ ± 0.94^ab^ | 1.94 x 10^5^ ± 0.49^a^ | 4.19 x 10^5^ ± 0.66^b^ | 0.1 | 0.2 | 0.04 | | |
| PS(42:4) | 2.68 x 10^3^ ± 1.15^a^ | 5.27 x 10^3^ ± 0.89^ab^ | 7.49 x 10^3^ ± 1.22^b^ | 0.2 | 0.008 | 0.2 | | |
| PS(32:3) | 5.36 x 10^3^ ± 1.70^c^ | 3.65 x 10^3^ ± 1.23^cd^ | 1.49 x 10^3^ ± 0.37^d^ | 0.7 | 0.07 | 0.2 | | |
| PS(38:4) | 1.57 x 10^4^ ± 0.60^a^ | 2.02 x 10^4^ ± 0.43^a^ | 3.52 x 10^4^ ± 0.31^b^ | 0.99 | 0.04 | 0.03 | | |
| PS(38:2) | 0.54 x 10^4^ ± 0.16^c^ | 1.59 x 10^4^ ± 0.35^bd^ | 0.46 x 10^4^ ± 0.08^ac^ | 0.08 | 0.9 | 0.04 | | |
| PS(42:8) | 0.76 x 10^3^ ± 0.21^ac^ | 2.49 x 10^3^ ± 0.56^bd^ | 1.05 x 10^3^ ± 0.20^c^ | 0.02 | 0.8 | 0.07 | | |

^1^ eicosapentaenoic acid

^2^ 3-carboxy-4-methyl-5-propyl-2-furanpropionic acid

^3^ pimelic acid

^4^ palmitoleic acid

^5^ eicosadienoic acid

**Supplementary Table 2.** Abundance of lipids in oocytes that differed between groups (NW; n=5, OB, n=7; OBD, n=6). Results are presented as mean ± SEM. Different superscripts within the same row indicate difference (^ab^, P < 0.05) or tendency for difference (^cd^, P < 0.1) between groups using one-way ANOVA with post-hoc Tukey’s multiple comparison tests.

| Lipid species | NW | OB | OBD | P value  (NW x OB) | P value  (NW x OBD) | P value  (OB x OBD) |
| --- | --- | --- | --- | --- | --- | --- |
| TG(50:1) | 4.95 x 10^5^ ± 0.36^a^ | 4.51 x 10^5^ ± 0.35^ab^ | 3.25 x 10^5^ ± 0.59^b^ | 0.7 | 0.04 | 0.1 |
| TG(50:1) | 1.66 x 10^5^ ± 0.17^cd^ | 1.71 x 10^5^ ± 0.19^c^ | 1.06 x 10^5^ ± 0.23^d^ | 0.99 | 0.1 | 0.06 |
| TG(54:1) | 4.73 x 10^4^ ± 0.73^cd^ | 6.07 x 10^4^ ± 0.90^c^ | 3.40 x 10^4^ ± 0.69^d^ | 0.5 | 0.5 | 0.06 |
| TG(58:9) | 1.28 x 10^4^ ± 0.31^a^ | 0.62 x 10^4^ ± 0.10^b^ | 0.57 x 10^4^ ± 0.13^b^ | 0.05 | 0.04 | 0.96 |
| TG(61:2) | 1.11 x 10^4^ ± 0.13^c^ | 1.86 x 10^4^ ± 0.13^d^ | 1.21 x 10^4^ ± 0.30^cd^ | 0.09 | 0.9 | 0.1 |
| TG(52:2) | 4.42 x 10^3^ ± 0.39^a^ | 3.29 x 10^3^ ± 0.49^ab^ | 2.33 x 10^3^ ± 0.57^b^ | 0.6 | 0.05 | 0.2 |
| TG(52:2) | 4.28 x 10^4^ ± 0.10^a^ | 3.37 x 10^4^ ± 0.50^ab^ | 2.43 x 10^4^ ± 0.42^b^ | 0.3 | 0.01 | 0.2 |
| TG(30:0) | 0.62 x 10^4^ ± 0.08^ac^ | 0.91 x 10^4^ ± 0.08^c^ | 1.80 x 10^4^ ± 0.45^bd^ | 0.7 | 0.02 | 0.07 |
| DG(24:0) | 3.31 x 10^3^ ± 0.40^ab^ | 2.80 x 10^3^ ± 0.38^a^ | 5.07 x 10^3^ ± 0.57^b^ | 0.6 | 0.04 | 0.2 |
| DPA^1^ | 0.66 x 10^4^ ± 0.15^c^ | 1.37 x 10^4^ ± 0.34^cd^ | 1.87 x 10^4^ ± 0.50^d^ | 0.3 | 0.09 | 0.6 |
| ODA^2^ | 0.93 x 10^2^ ± 0.15^ac^ | 1.24 x 10^2^ ± 0.16^c^ | 1.60 x 10^2^ ± 0.11^bd^ | 0.4 | 0.01 | 0.09 |
| OA^3^ | 0.77 x 10^4^ ± 0.19^c^ | 0.97 x 10^4^ ± 0.10^cd^ | 1.28 x 10^4^ ± 0.12^d^ | 0.8 | 0.06 | 0.1 |
| HDA^4^ | 1.88 x 10^3^ ± 0.12^c^ | 1.95 x 10^3^ ± 0.17^c^ | 2.40 x 10^3^ ± 0.14^d^ | 0.9 | 0.06 | 0.08 |
| SDA^5^ | 0.75 x 10^4^ ± 0.11^ac^ | 1.10 x 10^4^ ± 0.16^c^ | 1.83 x 10^4^ ± 0.33^bd^ | 0.5 | 0.01 | 0.07 |
| PC(46:5) | 2.82 x 10^3^ ± 0.18^a^ | 5.04 x 10^3^ ± 0.57^ab^ | 6.35 x 10^3^ ± 1.18^b^ | 0.2 | 0.02 | 0.4 |
| PE(42:2) | 6.65 x 10^3^ ± 1.42^a^ | 2.97 x 10^3^ ± 0.44^b^ | 4.77 x 10^3^ ± 1.24^ab^ | 0.05 | 0.4 | 0.4 |
| SM(16:1) | 1.87 x 10^4^ ± 0.14^a^ | 3.49 x 10^4^ ± 0.64^ab^ | 5.15 x 10^4^ ± 1.29^b^ | 0.4 | 0.05 | 0.3 |
| PA(40:3) | 0.22 x 10^4^ ± 0.02^c^ | 0.73 x 10^4^ ± 0.25^cd^ | 1.43 x 10^4^ ± 0.53^d^ | 0.6 | 0.07 | 0.3 |
| PS(34:0) | 2.86 x 10^3^ ± 0.30^a^ | 3.87 x 10^3^ ± 0.56^a^ | 6.72 x 10^3^ ± 1.08^b^ | 0.7 | 0.007 | 0.02 |

^1^ docosapentaenoic acid

^2^ octadecanedioic acid

^3^ oleic acid

^4^ heptadecenoic acid

^5^ stearidonic acid

**Supplementary Table 3.** Primer sequences of housekeeping gene (GAPDH) and genes of interest.

| Gene | Forward primer (5’- 3’) | Reverse primer (5’- 3’) | Product size (bp) | Gene Bank accession number |
| --- | --- | --- | --- | --- |
| GAPDH | GATCCCGCCAACATCAAATG | ACATTGGGGCATCAGCAGAA | 151 | NM_001163856.1 |
| CRAT | TCCCCAAGTCGGAGAAGCTG | CTGAGCGGATGGTGTCGGTA | 150 | XM_023629103.1 |
| CPT1B | CTTCCGCCAAACCCTGAAAC | ACTAGGCACGGGAAGCTTGG | 172 | XM_023631561.1 |
| PDK4 | CAGTTGACCCAGTCACCAATCA | CTTGGACCACTGCTGACACG | 180 | XM_001493731.6 |
| PDP1 | CCCGGAATCCCAGTCAGAAG | GGGGATGAGCAGCAGAGATG | 158 | XM_023648692.1 |
| LDHA | CACGTCAGCAGGAGGGAGAA | TAACGGAACCGGGCTGAATC | 223 | NM_001144880.1 |
| FASN | GGAAGACACGGCAGCTCTGA | TCCAGGCTCTGCTCCCTTTC | 201 | XM_023651730.1 |
| MTHFD2 | AATGTGGACGGCCTCCTTGT | GGGTAGCCGGTAACATGGAAT | 157 | XM_005599899.3 |
| SREBF1 | GCTCCTTGCAAACCCAGGTC | ACTCGCTGCCTTCACAGTGG | 206 | XM_023653674.1 |
| SREBF2 | ATCCAGCAGCAGGTGCAGAC | TCTGGACTGCAGCCATGACA | 217 | XM_005606691.3 |
| CCND2 | CCAGCCGAATGACAAGTTGC | CTTGGCCAGCAGGTCTACCA | 199 | NM_001309189.1 |
| LHCGR | CGGAGGAACCTCTCCCGACTAT | GGAGGTTGTCAAAGGCATTAGC | 151 | XM_023619111.1 |
| STAR | AGGCCATGGGAGAGTGGAAC | ATGCCAGCCAACACACAGGT | 181 | NM_001081800.3 |
| CYP11A1 | ACCGCCTCCTAGCAAGCAAC | ACTTCCTCCCGCAGCATCTC | 160 | NM_001082521.1 |
| CYP19A1 | ATGGGCATGCATGAGAATGG | CAACGCATTGGTGACCTCGT | 174 | NM_001081805.3 |
