# Supplementary material for: Follicular metabolic alterations are associated with obesity in mares and can be mitigated by dietary supplementation
Source: Sci Rep. 2024 Mar 30;14:7571. doi: 10.1038/s41598-024-58323-0 (PMC10981747; doi:10.1038/s41598-024-58323-0)
Supplement: Supplementary file 3 — Supplementary Information 3. [file 41598_2024_58323_MOESM3_ESM.docx]

Uncropped Western blots: SOD1 and SOD2, Figure 2h-i


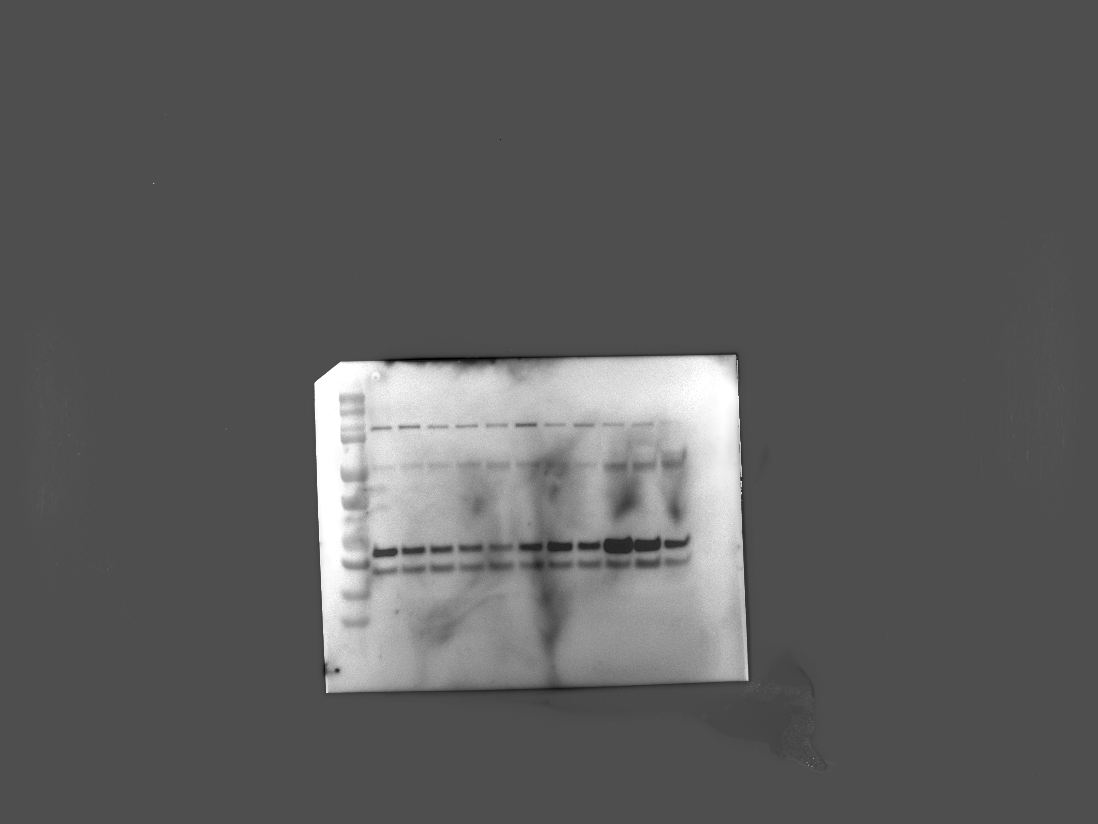


L 1 2 3 4 5 6 7 8 9 10 11

SOD2: 25 kDa

SOD1: 19 kDa


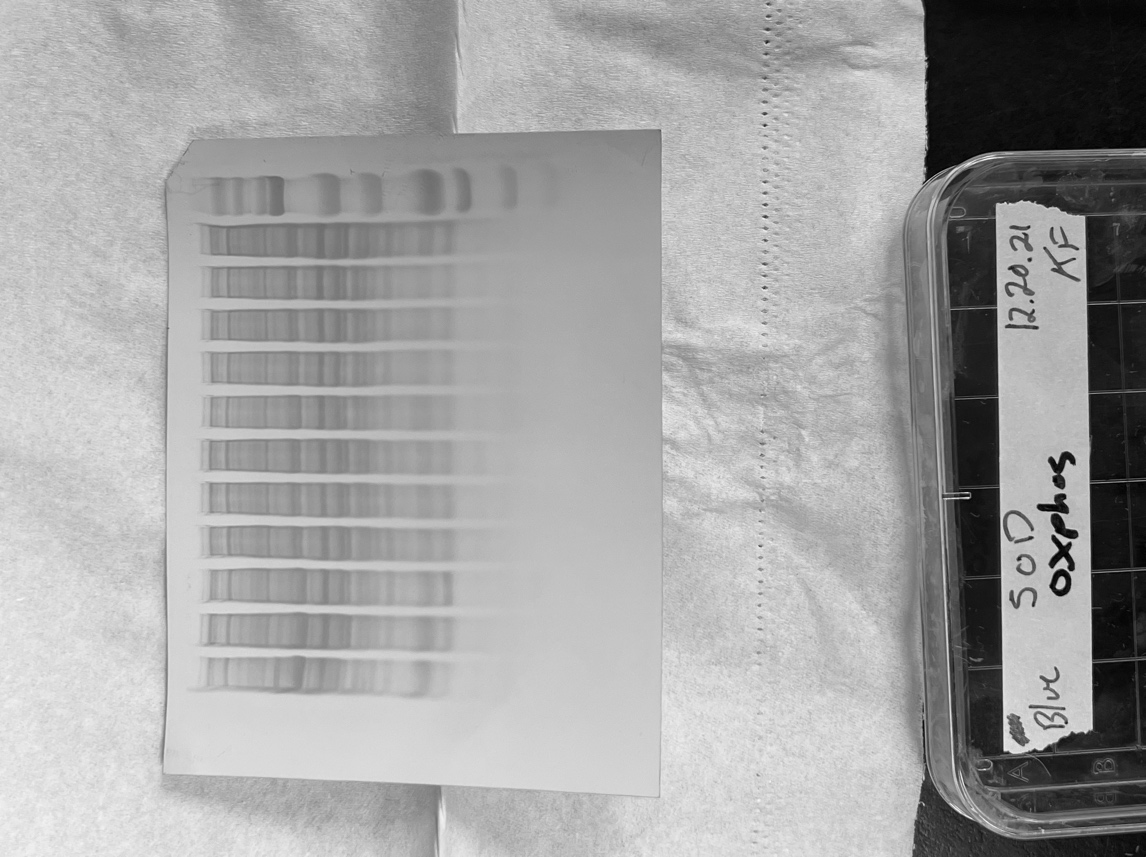


L 1 2 3 4 5 6 7 8 9 10 11

Amido Black total protein stain

L: Ladder

1: NW1

2: OB1

3: OBD1

4: NW2

5: OB2

6: OBD2

7: NW3

8: OB3

9: OBD3

10: NW4

11: OB4


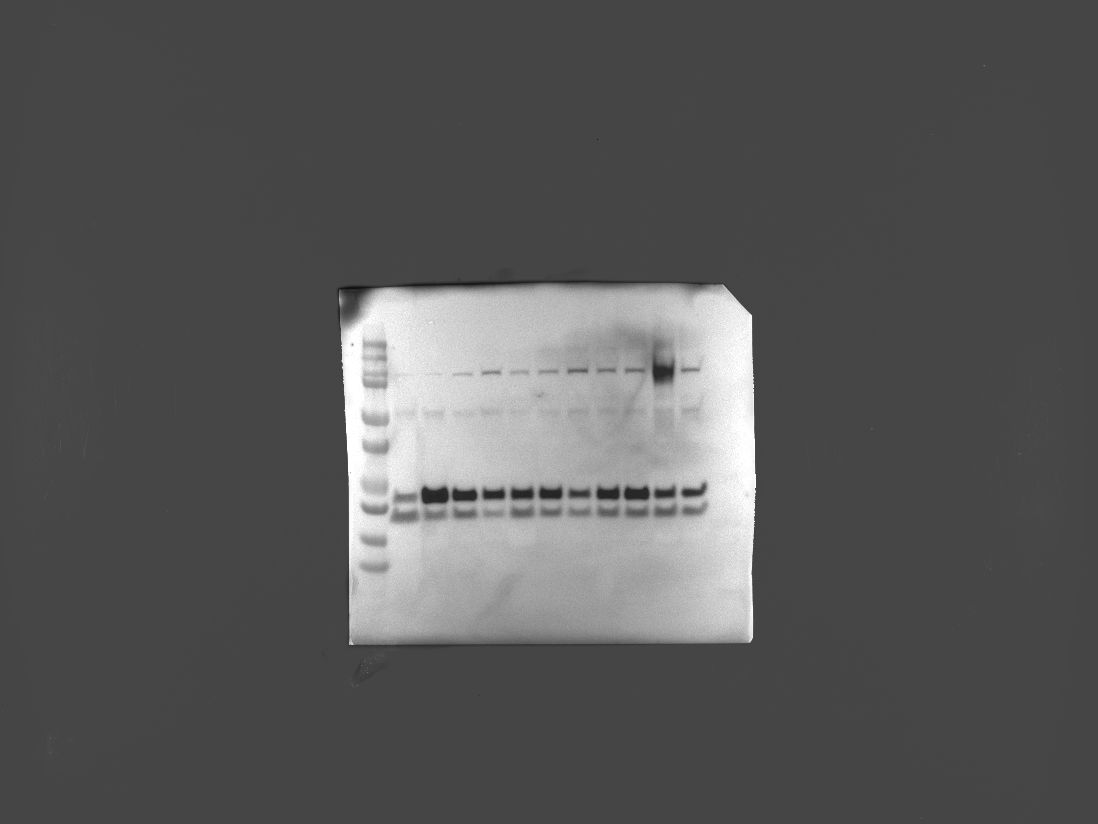


L 1 2 3 4 5 6 7 8 9 10 11

SOD2: 25 kDa

SOD1: 19 kDa


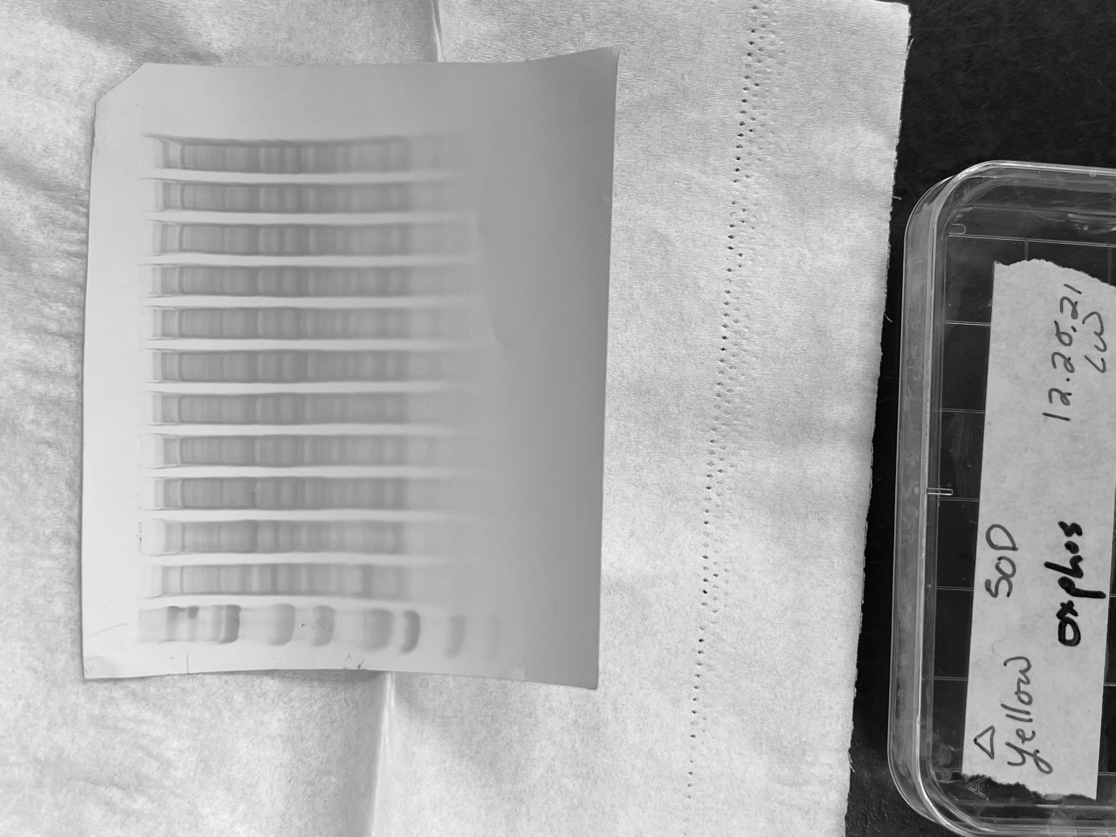


L 1 2 3 4 5 6 7 8 9 10 11

Amido Black total protein stain

L: Ladder

1: OBD4

2: NW5

3: OB5

4: OBD5

5: NW6

6: OB6

7: OBD6

8: NW1

9: OB7

10: OBD1

11: NW2

Uncropped Western blots: GPX1, Figure 2j


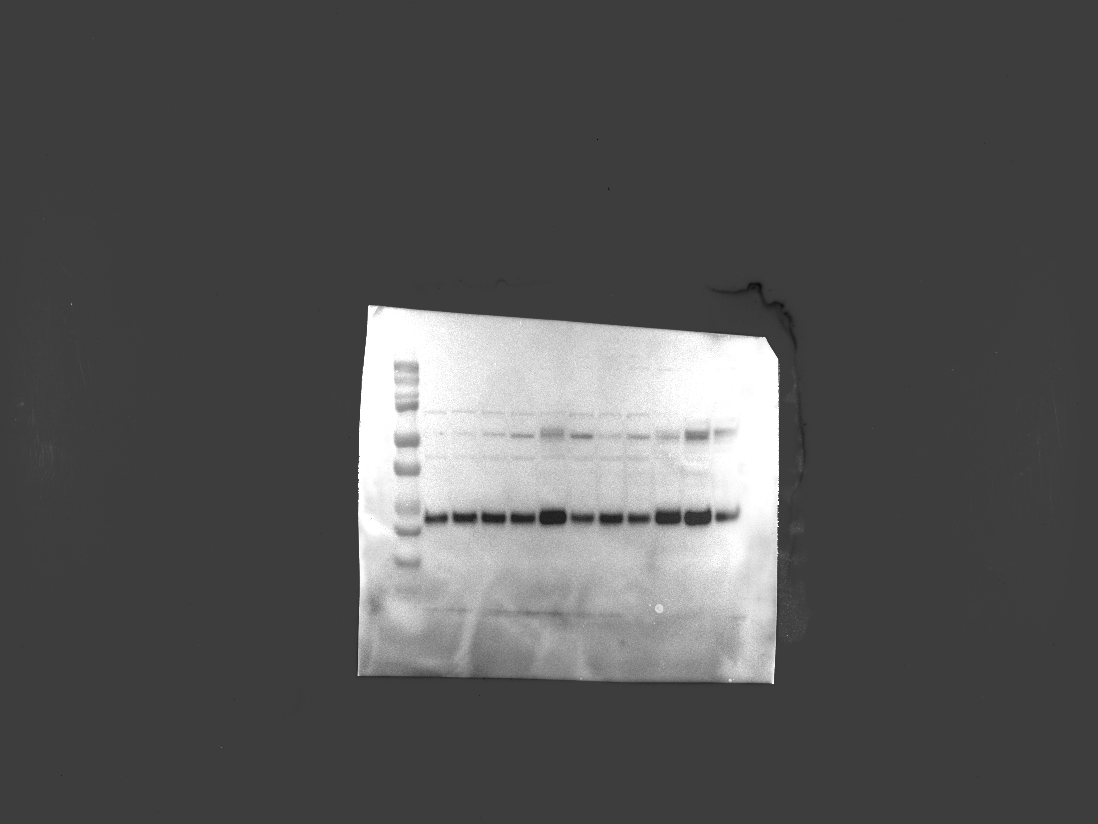


L 1 2 3 4 5 6 7 8 9 10 11

GPX1: 25 kDa


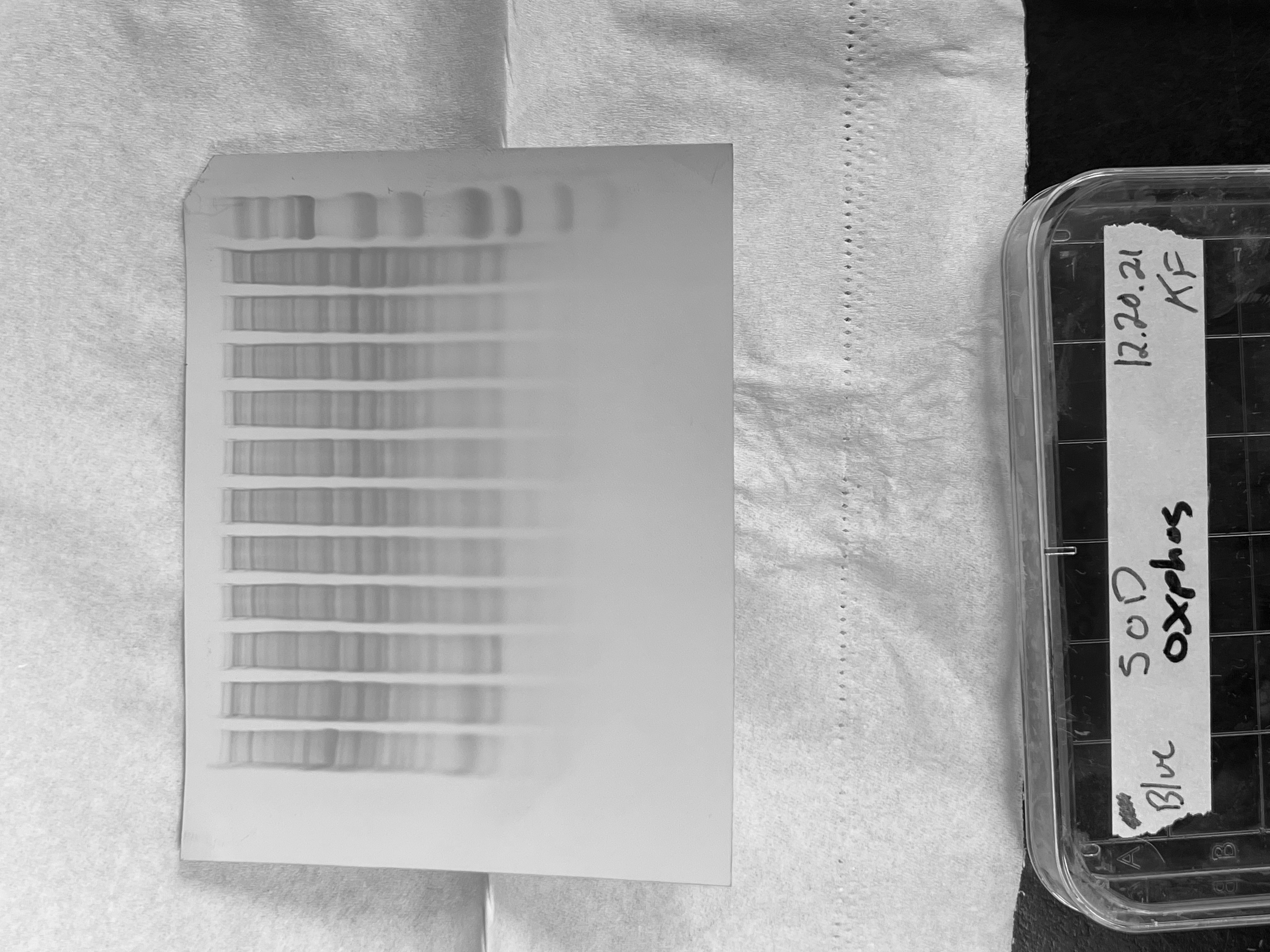


L 1 2 3 4 5 6 7 8 9 10 11

Amido Black total protein stain

L: Ladder

1: NW1

2: OB1

3: OBD1

4: NW2

5: OB2

6: OBD2

7: NW3

8: OB3

9: OBD3

10: NW4

11: OB4


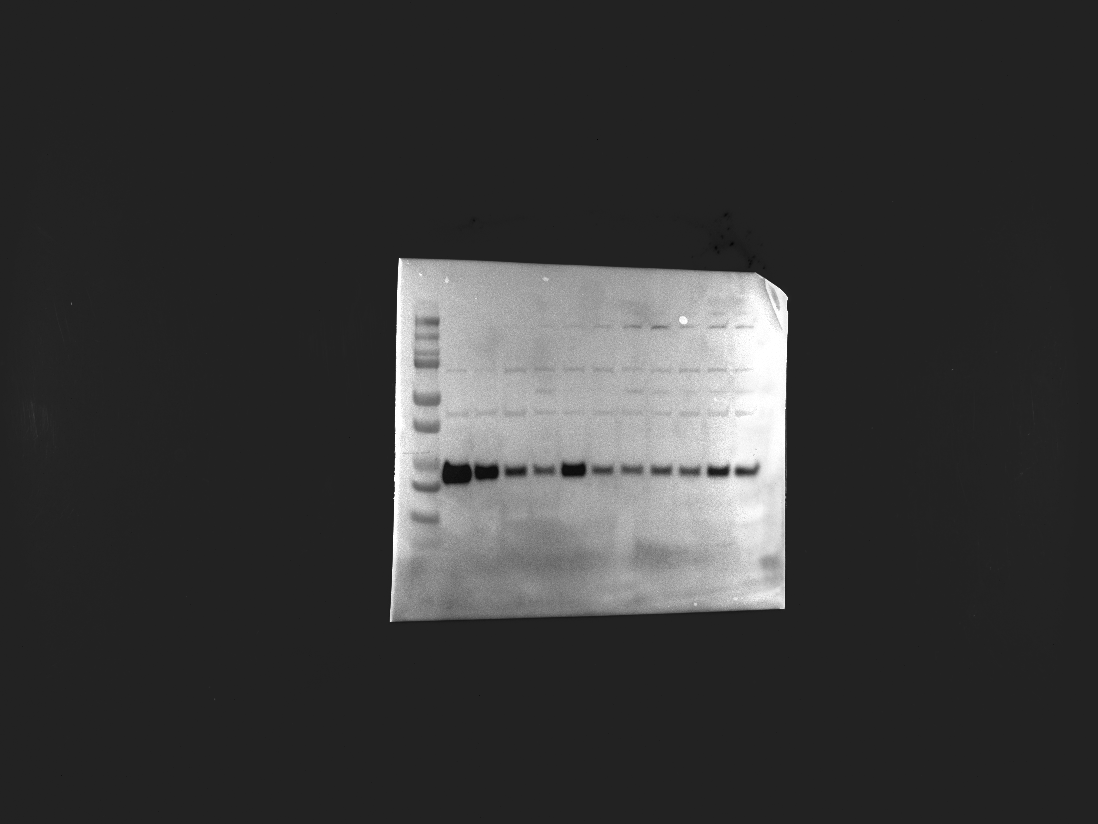


L 1 2 3 4 5 6 7 8 9 10 11

GPX1: 25 kDa


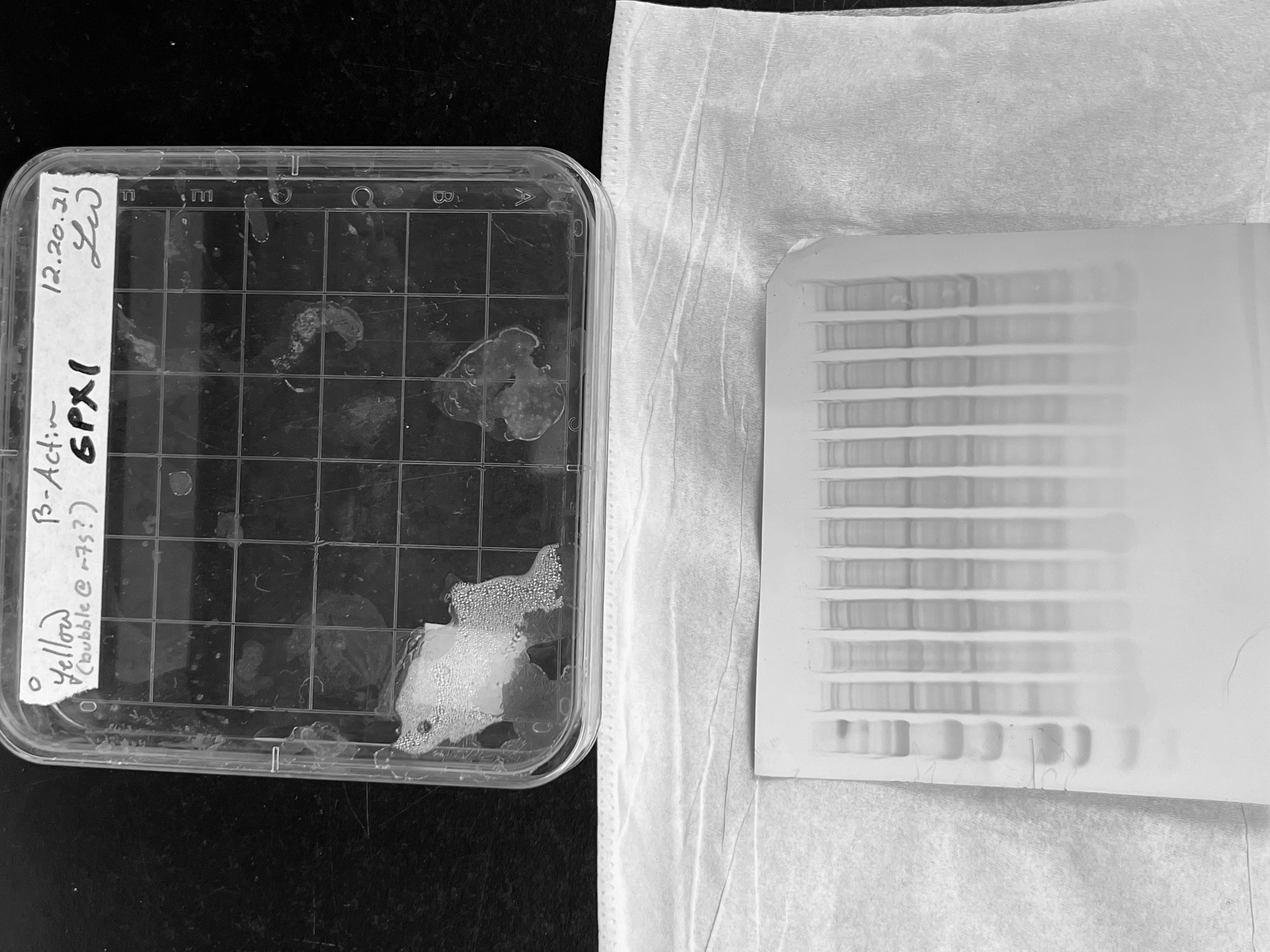


L 1 2 3 4 5 6 7 8 9 10 11

Amido Black total protein stain

L: Ladder

1: OBD4

2: NW5

3: OB5

4: OBD5

5: NW6

6: OB6

7: OBD6

8: NW1

9: OB7

10: OBD1

11: NW2

Uncropped Western blots: OXPHOS, Figure 2b-f


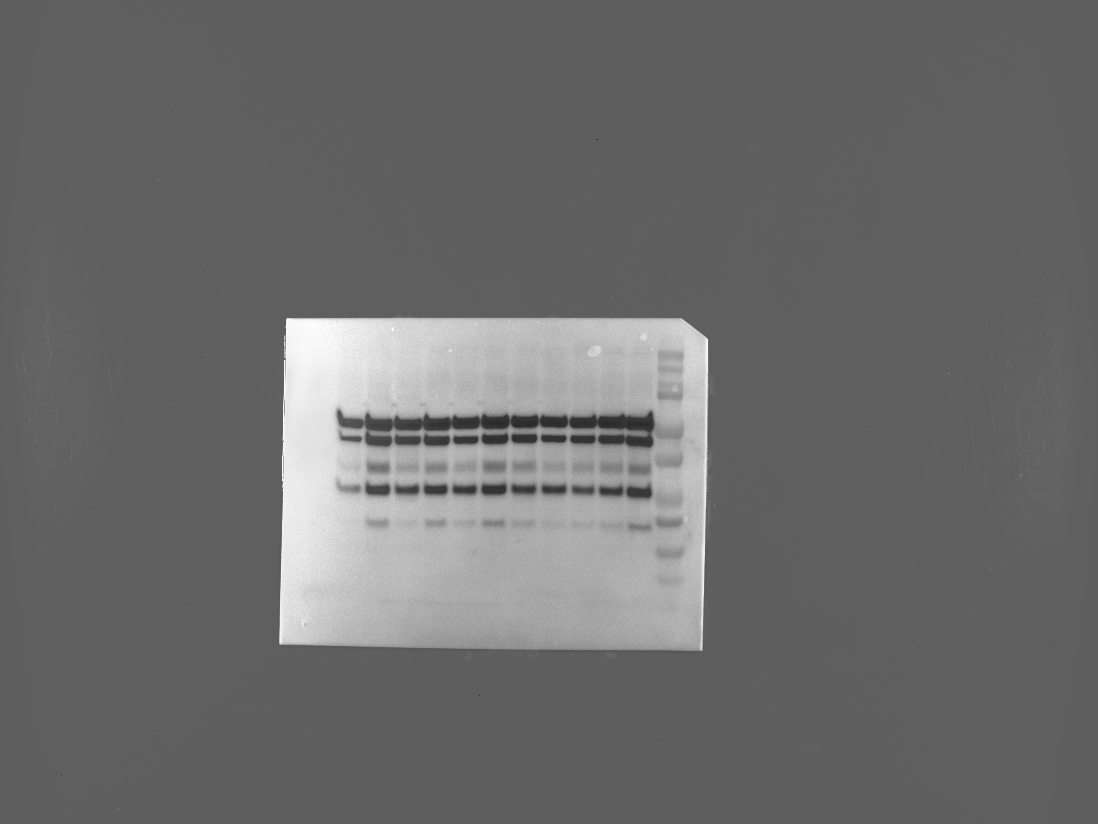


L 1 2 3 4 5 6 7 8 9 10 11

CV: 58 kDa

CIII: 45 kDa

CIV: 35 kDa

CII: 30 kDa

CI: 18 kDa


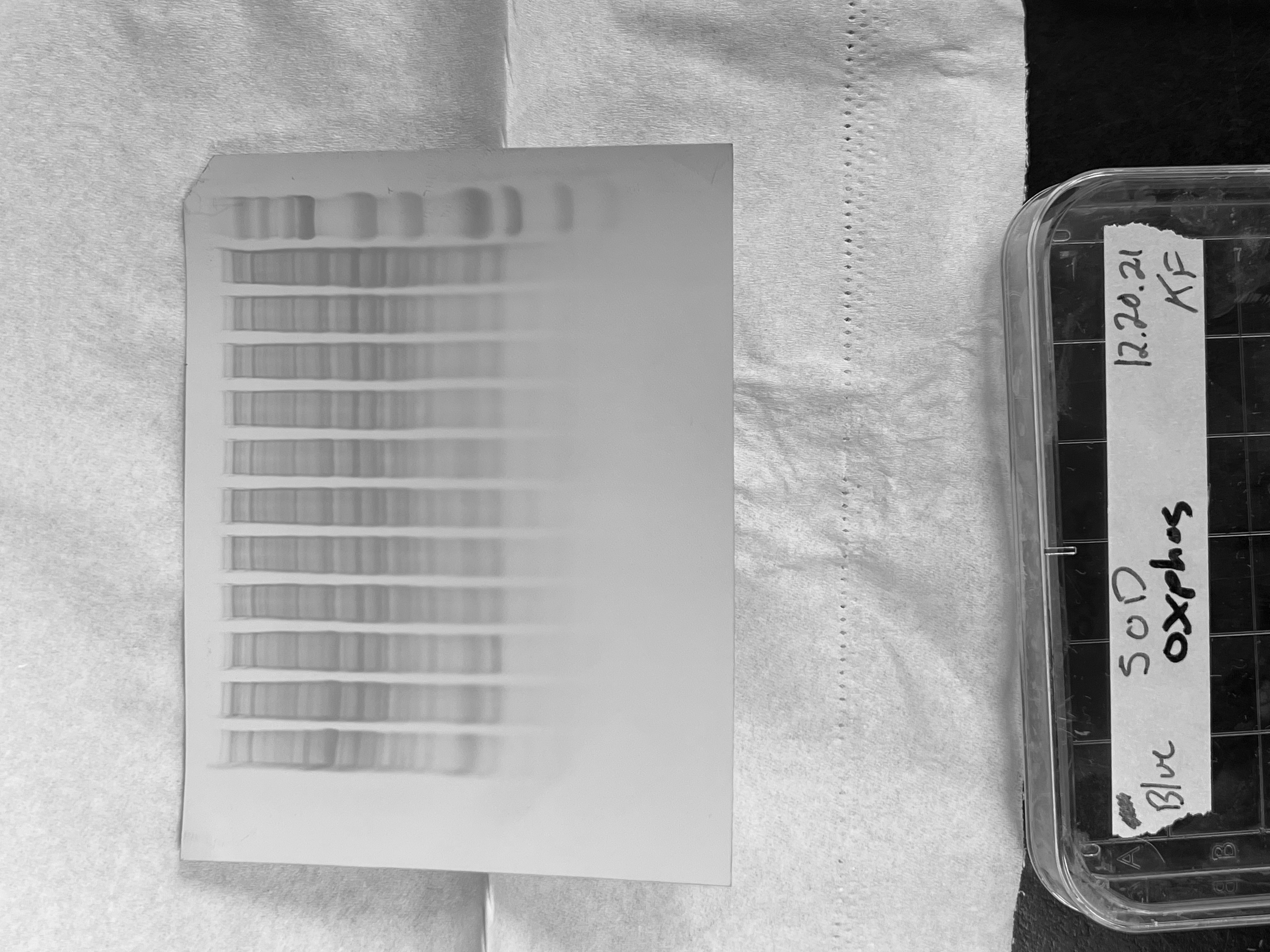


L 1 2 3 4 5 6 7 8 9 10 11

Amido Black total protein stain

L: Ladder

1: NW1

2: OB1

3: OBD1

4: NW2

5: OB2

6: OBD2

7: NW3

8: OB3

9: OBD3

10: NW4

11: OB4


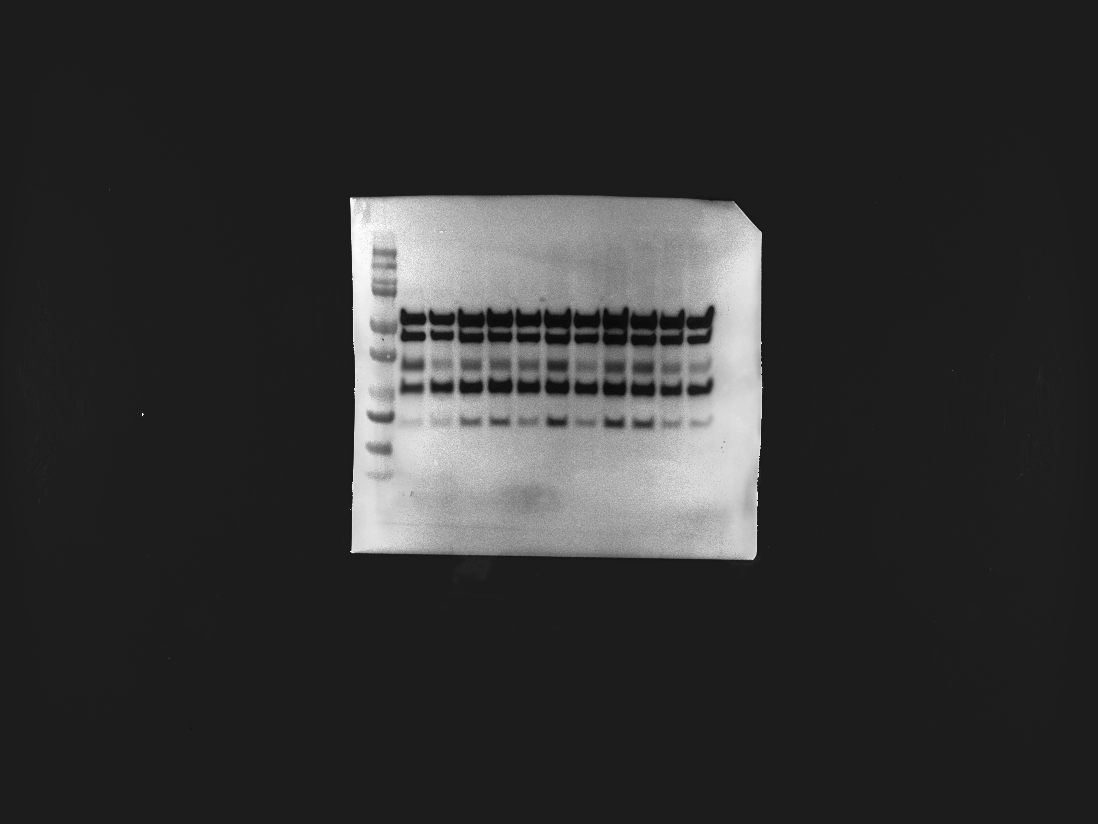


L 1 2 3 4 5 6 7 8 9 10 11

CV: 58 kDa

CIII: 45 kDa

CIV: 35 kDa

CII: 30 kDa

CI: 18 kDa


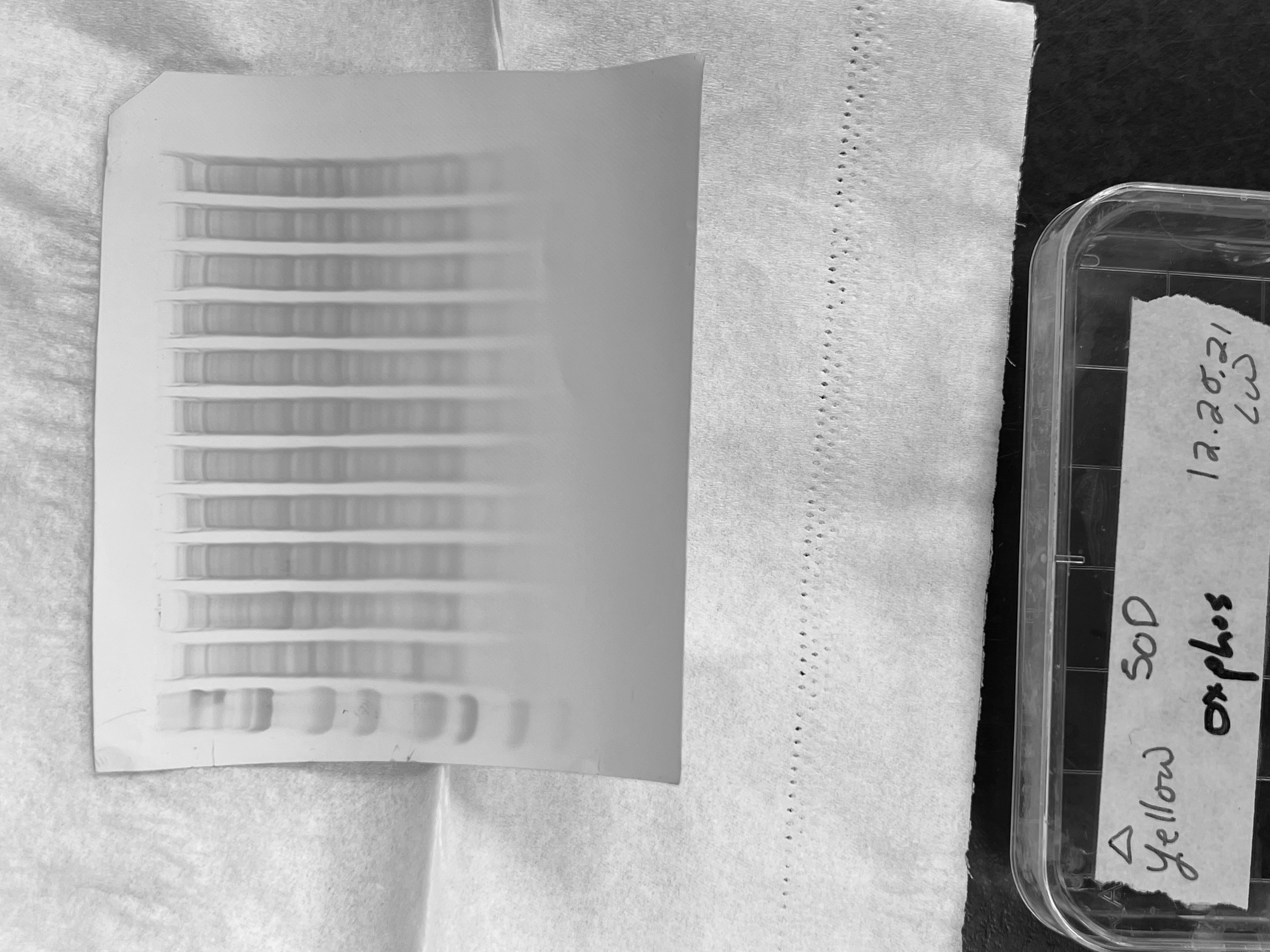


L 1 2 3 4 5 6 7 8 9 10 11

Amido Black total protein stain

L: Ladder

1: OBD4

2: NW5

3: OB5

4: OBD5

5: NW6

6: OB6

7: OBD6

8: NW1

9: OB7

10: OBD1

11: NW2
